# MAPK inhibitors dynamically affect melanoma release of immune NKG2D-ligands, as soluble protein and extracellular vesicle-associated

**DOI:** 10.3389/fcell.2022.1055288

**Published:** 2023-01-16

**Authors:** Silvia López-Borrego, Carmen Campos-Silva, Amaia Sandúa, Tamara Camino, Lucía Téllez-Pérez, Estibaliz Alegre, Alexandra Beneitez, Ricardo Jara-Acevedo, Annette Paschen, María Pardo, Álvaro González, Mar Valés-Gómez

**Affiliations:** ^1^ Department of Immunology and Oncology, National Center for Biotechnology (CNB), Spanish National Research Council (CSIC), Cantoblanco, Madrid, Spain; ^2^ University of Navarra, Pamplona, Navarre, Spain; ^3^ Health Research Institute of Santiago de Compostela (IDIS), Santiago de Compostela, Galicia, Spain; ^4^ Immunostep, Salamanca, Spain; ^5^ Clinic for Dermatology University Hospital of Essen, Essen, North RhineWestphalia, Germany

**Keywords:** metastatic melanoma, targeted cancer therapy, immunomodulation, immune evasion, extracellular vesicles, metalloproteases

## Abstract

Metastatic melanoma presents, in many cases, oncogenic mutations in BRAF, a MAPK involved in proliferation of tumour cells. BRAF inhibitors, used as therapy in patients with these mutations, often lead to tumour resistance and, thus, the use of MEK inhibitors was introduced in clinics. BRAFi/MEKi, a combination that has modestly increased overall survival in patients, has been proven to differentially affect immune ligands, such as NKG2D-ligands, in drug-sensitive vs. drug-resistant cells. However, the fact that NKG2D-ligands can be released as soluble molecules or in extracellular vesicles represents an additional level of complexity that has not been explored. Here we demonstrate that inhibition of MAPK using MEKi, and the combination of BRAFi with MEKi *in vitro*, modulates NKG2D-ligands in BRAF-mutant and WT melanoma cells, together with other NK activating ligands. These observations reinforce a role of the immune system in the generation of resistance to directed therapies and support the potential benefit of MAPK inhibition in combination with immunotherapies. Both soluble and EV-associated NKG2D-ligands, generally decreased in BRAF-mutant melanoma cell supernatants after MAPKi *in vitro*, replicating cell surface expression. Because potential NKG2D-ligand fluctuation during MAPKi treatment could have different consequences for the immune response, a pilot study to measure NKG2D-ligand variation in plasma or serum from metastatic melanoma patients, at different time points during MAPKi treatment, was performed. Not all NKG2D-ligands were equally detected. Further, EV detection did not parallel soluble protein. Altogether, our data confirm the heterogeneity between melanoma lesions, and suggest testing several NKG2D-ligands and other melanoma antigens in serum, both as soluble or vesicle-released proteins, to help classifying immune competence of patients.

## Introduction

Metastatic melanoma is an aggressive type of skin cancer whose incidence has been growing in Northern Europe, North America and Australia over the last decades (Matthews et al., 2017; Siegel et al., 2020). Until quite recently, it was a fatal disease with 1-year survival rate of only 27% of patients, when treated with chemotherapy, such as dacarbazine (Davis et al., 2019). The discovery that most melanoma lesions have mutations in members of the mitogen-activated protein kinase (MAPK) pathway led to the use of new targeted drugs which improved melanoma patient life expectancy. Recently, the use of immunotherapies has further improved patient prognosis, leading to 5-year survival rates in over 30% of patients (Kuske et al., 2018). Despite the clear improvement that new therapies have represented for melanoma patients, a lack of response still occurs in many patients and the appearance of drug-resistant tumours is also a frequent event, highlighting the need for improved therapeutic regimes, based on the knowledge of the mechanisms causing treatment failure.

The MAPK signaling cascade includes kinases such as RAS, RAF, MEK and ERK, and regulates cell proliferation and survival in response to growth factors. This pathway is mutated in approximately 70% of melanoma (Ikawa et al., 1988). The most common BRAF^V600E/K^ mutations (Val to Glu or Lys at position 600) are present in 40%–50% of melanoma lesions, leading to the constitutive activation of the MAPK pathway, and promoting uncontrolled proliferation and cell survival (Davies et al., 2002; Gray-Schopfer et al., 2007). The first inhibitor of mutated BRAF (BRAFi), approved by the FDA (2011) and EMA (2012), was vemurafenib (PLX4032), and other drugs targeting BRAF were later approved, like dabrafenib (GSK2118436), with similar results in melanoma patients (Chapman et al., 2011). Although 50% of vemurafenib-treated patients present a generally rapid response, with a mean Overall Survival (OS) of 15.9 months (Sosman et al., 2012), progression occurs generally after around 6 months, due to the emergence of tumour drug resistance.

Several mechanisms were proposed for tumour-BRAFi resistance, including the reactivation of downstream kinases in the MAPK cascade, for instance, MEK or ERK, as well as the activation of the PI3K/mTOR pathway, also involved in cell survival and proliferation (Knispel et al., 2018). These data led to the use of trametinib, an inhibitor of MEK1 and MEK2 (MEKi), which combined with BRAFi resulted in higher patient response rates and prolonged Progression-Free Survival (PFS) to 11 months (Robert et al., 2014). However, other mechanisms related to the immune response were also described as possible reasons for treatment failure. Tumours from BRAFi-treated patients initially show an increase in melanoma differentiation antigens, such as tyrosinase-related protein 1 (TYRP-1) and the melanoma-associated antigen (MART-1/Melan-A), recognized by T cells (Boni et al., 2010; Frederick et al., 2013; Bradley et al., 2015; Pieper et al., 2018), which are associated with an increase in the infiltration of CD8^+^ T cells that could contribute to anti-tumor immunity. However, as treatment progresses, these effects are reverted, supporting that combination of MAPKi with other therapies would be more beneficial than MAPKi as *solo* treatment. In consequence, recently, many clinical trials have tested the effect of combining targeted therapy and immune checkpoint blockade [reviewed in (Yu et al., 2019; Ziogas et al., 2021)]. The first results reported a certain benefit, but high rates of severe adverse effects and non-responders are still a problem.

A crucial feature for immune recognition of tumour cells is surface expression of ligands for NKG2D, an activating immune receptor expressed by αβ CD8^+^ T cells, γδ T cells and NK cells (Groh et al., 1999; Smyth et al., 2005; Guerra et al., 2008). The involvement of the NKG2D receptor-ligand system in cancer elimination has been long known, as well as the existence of evasion mechanisms affecting these molecules that result in cancer progression (Wu et al., 2009). In fact, the presence of NKG2D-ligands (NKG2D-L) in patient sera, together with the decrease of NKG2D receptor on effector lymphocytes, has been associated to worse prognosis of patients suffering many different types of cancer, both solid and haematological tumours [(Campos-Silva et al., 2022b) and references therein].

In humans, NKG2D-L include two families of MHC-I-related proteins: MHC class I polypeptide-related chains A and B (MICA/B) and UL16-binding proteins (ULBP1-6). These proteins are usually expressed at the cell surface of stressed cells, such as pathogen-infected or transformed cells, promoting cytotoxic NK functions both *in vivo* and *in vitro* (Mistry and O'Callaghan, 2007; Gonzalez et al., 2008; Spada et al., 2015; Campos-Silva et al., 2022b). As many other cancer cell types, melanoma cells can express different NKG2D-L and their presence has been related with their lysis not just by NK cells, but also T cells (Casado et al., 2009; Lakshmikanth et al., 2009; Paschen et al., 2009; Schwinn et al., 2009). However, an extra level of complication arises from the fact that NKG2D-L can be released from the cell surface either by metalloprotease cleavage or recruited to extracellular vesicles (EVs) [reviewed in (Fernandez-Messina et al., 2012; Campos-Silva et al., 2018)]. Exosomes are a subset of EVs, ranging from 30 to 150 nm in diameter, commonly originated from invaginations of endocytic compartments and released by fusion of multivesicular body organelles with the plasma membrane (Yáñez-Mó et al., 2015; Mannavola et al., 2019). The endocytic origin of EVs containing NKG2D-L has not been demonstrated, for this reason we refer to NKG2D-L-containing vesicles as EVs, and not as exosomes (Thery et al., 2018; Witwer et al., 2021).

Membrane-bound NKG2D-L are potent activators of NK cells by ligation with the NKG2D receptor, which signals through the PI3K pathway, activating the lytic machinery of effector cells (Billadeau et al., 2003; Upshaw et al., 2006). However, soluble (sNKG2D-L) and EV-NKG2D-L inhibit cytotoxicity against tumours and are associated with NKG2D receptor downmodulation, causing a loss of effector function. These effects have been studied both *in vitro* (Ashiru et al., 2010; Fernandez-Messina et al., 2010) and in murine models, in which the lack of NKG2D led to a higher tumor risk (Guerra et al., 2008). Further, transgenic mice expressing a shedding-resistant MICA avoided tumor progression (Groh et al., 2002). Recently, antibodies targeting soluble MIC or blocking its release have been designed for their use in therapy, the latest with promising results in murine and macaque models (Badrinath et al., 2022). However, the effect of EV-recruited NKG2D-L has not been assessed in this system.

The role of NKG2D and its ligands has also been highlighted in clinical trials with immune check point inhibitors. For example, the absence of soluble ULBP1 and MICB were related with better clinical outcome for melanoma patients treated with anti-CTLA-4 or anti-PD-1 antibodies (Maccalli et al., 2017). Hence, release of NKG2D-L has been demonstrated to constitute a major mechanism for tumor cell evasion and many data suggest that these molecules could mirror patient immune competency. In this sense, it is still necessary to evaluate carefully the immunomodulation effects induced by targeted therapies on the NKG2D system.

In order to understand the mechanisms involved in tumour immune evasion and the changes exerted by targeted therapy combinations, here we characterized the different biological routes followed by NKG2D-L, after treatment with single MAPKi or the combination of two inhibitors, vemurafenib (BRAFi) and trametinib (MEKi). Our laboratory and others have previously described the effect of vemurafenib on surface expression of NKG2D-L (López-Cobo et al., 2017; Frazao et al., 2017). However, even though the effects of BRAFi/MEKi combination on melanoma cell immunogenicity has been previously studied (Pieper et al., 2018; Frazao et al., 2020; Harbers et al., 2021), little is known about the impact of single MEKi or combined BRAFi/MEKi on the NKG2D system on drug sensitive cells. In addition, the impact of MAPK inhibition on vesicle-released ligands was not studied. Here, we hypothesized that, if NKG2D-L are affected by MAPKi, it should be possible to test the release of either soluble or EV-associated NKG2D-L to follow-up the dynamics of immunosuppression events induced by targeted therapies. To test this, we analysed secreted NKG2D-L in serum and plasma samples from melanoma patients obtained at different time points after starting treatment with these drugs.

Here we describe that *in vitro* treatment of BRAF^V600E^-mutant melanoma cells with vemurafenib (BRAFi) and trametinib (MEKi), as *solo* or combined therapies, leads to a downmodulation of surface NK activating ligands (NKG2D and DNAM-1-ligands), which paralleled with a decrease in total MICA protein levels in cell lysates. These results are in line with previous data, described for vemurafenib treatment, and suggest that NKG2D-L expression could affect NK cell recognition. Soluble and EV-associated NKG2D-L also decreased after the treatment *in vitro*, and changes in these secreted proteins were observed in serum and plasma samples from melanoma patients during treatment with MAPKi. However, different NKG2D-L and their diverse biochemical forms had different profiles in biological samples. These data support the idea that testing NKG2D-L as putative biomarkers of immune evasion events requires complete and personalized studies. Thus, a rationale for the design of alternative drug combination strategies could be envisioned for each situation, for example, enhancing surface NKG2D-L.

## Materials and methods

### Reagents

Unless otherwise stated, all chemicals were purchased from Merck & Co. (Kenilworth, New Jersey, United States). Primers were purchased from Sigma (Sigma-Aldrich, St. Louis, MO, United States). Antibodies used for different techniques are listed in Supplementary Table S1.

### Cell lines and culture conditions

The human metastatic melanoma cell lines Ma-Mel-86c, −86f, −55 and −103b were obtained as previously described (Ugurel et al., 2007; Zhao et al., 2016). Ma-Mel-86c, −86f and −55 contain the BRAF^V600E^ mutation, whereas -103b cells are wild-type for BRAF. Moreover, MICA alleles are differentially expressed in these cell lines (López-Cobo et al., 2017). Lung cancer cell line H3122 was purchased from ATCC. All cells were cultured in RPMI-1640 culture medium, supplemented with 10% fetal bovine serum (FBS), 1 mM l-glutamine, 1 mM sodium pyruvate, 0.1 mM non-essential amino-acids, 10 mM Hepes, 100 U/ml penicillin and 100 U/ml streptomycin (Biowest, Nuaillé, France) (complete medium) at 37°C, with humidified atmosphere of 5% CO_2_, and subcultured twice a week in a T-75 flask. Cells were regularly tested for *mycoplasma* contamination.

### Cell culture drug treatments

Metastatic melanoma cells were treated with either vemurafenib (PLX4032) (Selleckhem, Houston, Texas, United States) or trametinib (MedChemExpress, Monmouth Junction, New Jersey, United States) dissolved in dimethyl sulfoxide (DMSO) and used at 1 μM and 50 nM, respectively, or the combination of both (as previously described in (López-Cobo et al., 2017; Benito-Jardon et al., 2019). Incubation with the same concentration of DMSO (1:1000) was used as vehicle control. Cells were incubated for 24 or 48 h in medium with these treatments as explained for each experiment and the number of cells recovered in each condition was evaluated (Supplementary Table S2). In previous studies, cell proliferation reduction and ERK1/2 inactivation were demonstrated under the same experimental conditions (López-Cobo et al., 2017; Benito-Jardon et al., 2019). Here, a reduction in cell proliferation with respect to the untreated control condition (vehicle control) was taken as evidence of the MEK1/2-ERK1/2 pathway suppression (Supplementary Table S2).

### Melanoma patient sera/plasma

Ethical principles established in the Declaration of Helsinki were followed. Patients (or their representatives) were informed about the study and gave a written informed consent. Samples were obtained at Clínica Universidad de Navarra. The Ethics Committee of Clínica Universidad de Navarra did not raise any concern about ethical issues and approved the project (internal project approval number 2022.063).

Samples from 29 melanoma patients and 16 non-cancer patients were used. Demographic and clinical data from these cohorts are available in Tables 1, 2. Blood was collected from each subject either in a 5 ml EDTA [BD Vacutainer^®^ K2E (EDTA), BD Biosciences, Franklin Lakes, New Jersey, United States] or serum tube (BD Vacutainer^®^ SSTTM II Advance, BD Biosciences, Franklin Lakes, New Jersey, United States) and, after centrifugation 10 min at 2000 *x g* at RT, supernatants were recovered and frozen at −80°C until test. Samples were thawed and directly used in experiments. For repetitions, aliquots were made and re-frozen at -80°C, to avoid further freeze–thawing cycles. For comparison, non-cancer donor samples were analysed, however, since these individuals attended the hospital for a blood test, other pathologies cannot be ruled out.

**TABLE 1:** Metastatic melanoma patients treated with MAPKi. Demographic and pathological data.

Characteristics		Metastatic melanoma (*N* = 22)	Patient number	Non-cancer patients (*N* = 6)
		Number (%)		Number (%)
*Age*	Mean	50.31		41.75
	Min-Max	33–82		24–72
*Sex*	Male	14 (63.63)		4 (66.66)
	Female	8 (36.36)		
	N.A.			2 (33.33)
*Clinical Stage*	IIIA	1 (4.54)		
	IIIB	1 (4.54)		
	IV	20 (90.9)		
*Treatment*	BRAFi			
	- Vemurafenib	14 (63.6)	1–4, 7–10, 12–15, 17,18	
	- Dabrafenib	4 (18.18)	5, 6, 11, 16	
	MEKi			
	- Cobimetinib	0 (0)		
	- Trametinib	0 (0)		
	- Binimetinib	1 (4.54)	23	
	Combined BRAFi with MEKi			
	- Vemurafenib + (cobimetibib/placebo)	2 (9.09)	19, 20	
	- (Dabrafenib/placebo) + (trametinib/placebo)	1 (4.54)	24	

**TABLE 2:** Metastatic melanoma patients treated with MAPKi in combination. Demographic and pathological data.

Characteristics		Metastatic melanoma (*N* = 7)	Corresponding patient number	Non-cancer patients (*N* = 10)
		Number (%)		Number (%)
*Age*	Mean	52		46
	Min-Max	33–65		22––67
*Sex*	Male	4 (57.14)		5 (50)
	Female	3 (42.85)		5 (50)
*Clinical Stage*	IIIA	1 (14.28)		
	IIIB	1 (14.28)		
	IV	5 (71.42)		
*Treatment*	BRAFi			
	- Vemurafenib			
	- Dabrafenib	1 (14.28%)	35	
	MEKi			
	- Cobimetinib			
	- Trametinib			
	- Binimetinib			
	Combined BRAFi with MEKi			
	- Vemurafenib + cobimetibib	0 (0%)		
	- Dabrafenib + trametinib	2 (28.57%)	36, 37	
	- Dabrafenib+ (trametinib or placebo)	2 (28.57%)	31, 32	
	- (Dabrafenib or placebo) + (trametinib or placebo)	2 (28.57%)	33, 34	
		

### Flow cytometry

Following 24 and 48 h of treatment with vemurafenib, trametinib, or the combination of both, 1 x 10^5^ cells per condition were detached with PBE [Phosphate-Buffered Saline (PBS, 10 mM Na_2_HPO_4_, 1.8 mM KH_2_PO_4_, 137 mM NaCl, 2.7 mM KCl), 0.1% bovine serum albumin (BSA), 0.2 mM EDTA] and stained for flow cytometry analyses. Melanoma cells were labeled with the mouse monoclonal primary antibodies listed in Supplementary Table S1, diluted in PBA [PBS, 1% BSA, 0.1% sodium azide]. For detection of surface proteins, 0.02 μg/μL of phycoerythrin-labelled goat anti-mouse Ig (Dako, Brüsseler, Jena, Germany) was used as secondary antibody. In some experiments, cell death was determined by the analysis of DAPI (D9542, Sigma) staining, used at 5 μg/ml. Samples were acquired using the Cytomics FC500 or Gallios Flow Cytometer (Beckman Coulter, Brea, California, United States) and experiments were analyzed using Kaluza software (Beckman Coulter). Mean Fluorescence Intensity (MFI) was obtained for each antibody and Relative Fluorescence Intensity (RFI) was calculated as: RFI = MFI_antibody_/MFI_isotype control_.

#### Enzyme linked ImmunoSorbent assay (ELISA)

For the detection of soluble NKG2D-L, sandwich ELISA were performed. Plates were coated with anti-human MICA, MICB, ULBP1, ULBP2/5/6, and ULBP3 (Supplementary Table S1), diluted in Borate-Buffered Saline (BBS) at 5 μg/ml. After incubation for 16 h at 4°C under humidity, plates were washed with PBS-containing 0.05% Tween-20 (PBS-T) and blocked with 1% casein-PBS at pH = 7.4 (Biorad, Hercules, California, United States) for 2 h at 37°C. Standard dilutions were prepared from recombinant human MICA (1300-MA), MICB (1599-MB-050), ULBP1 (1380-UL), ULBP2 (1298-UL) and ULBP3 (1517-UL) (R&D) in 1% casein-PBS. Supernatants collected after the treatment of Ma-Mel cells for 24 and 48 h, which were centrifuged at 200 *x g* to discard floating cells, or plasma and serum samples diluted 1:2 in 1% casein-PBS, were used as test samples. Standards and samples were incubated 18 h at 4°C. For the detection, plates were incubated for 1 h at RT with biotinylated antibodies diluted in 1% casein-PBS. This step was followed by incubation with streptavidin-HRP (0.25 μg/ml, BioLegend, San Diego, California, United States) for 1 h at RT and the addition of TMB (3,3′,5,5′-Tetramethylbenzidine) substrate (1-Step Ultra TMB-ELISA Substrate Solution (Thermo Scientific, Waltham, MA, United States). Absorbance was measured at 450 nm in a Thermo Scientific™ Multiskan™ FC Filter-based Microplate Photometer.

For analysis of proteins in sera or plasma-derived EVs, a sandwich containing antibodies directed to different EV-related molecules was designed (see cartoons in Figure 6; Supplementary Figure S4). Capture antibodies, anti-human MICA and anti-human CD63 (or IgG1 isotype control) were coated, as above, in BBS. For washes, instead of PBS-T, HEPES-buffered saline (HBS: 10 mM HEPES pH 7.2, 150 mM NaCl), containing 0.05% Tween-20, was used. To avoid metalloprotease cleavage of MICA on serum EVs, either 6 mM EDTA was added to serum samples, or incubation was carried at 4°C. For detection of EV-bound proteins, biotinylated anti-CD9 or anti-CD81 secondary antibodies were used. The different combinations performed are described in figure legends. As a positive control for EV detection, both Ma-Mel-86c- and H3122-derived EVs (4 x 10^7^ EV/μL) were added, being the former ones positive for MICA detection and the latter ones used as a negative control.

### EV preparations

Metastatic melanoma cells were incubated for 48 h in medium containing 1% EV-free FBS with either vemurafenib, trametinib or the combination. 2 × 15 cm cell culture dishes were used per treatment. Cells were lysed and used for Western Blot analyses and supernatants were collected and immediately centrifuged twice at 200 *x g* for 5 min, to pellet cells. To discard cell debris and fragments, two sequential centrifugations at 500 *x g* for 10 min were performed. Large vesicles were removed by centrifugation (Sorvall RC5C centrifuge, rotor Sorvall SS-34) at 10,000 *x g* for 30 min, and supernatants were finally ultracentrifuged (Optima L-100 XP Ultracentrifuge and rotor type SW28Ti) (Beckman Coulter) at 100,000 *x g* for 2 h, without brake. Differential centrifugations were all carried out at 4°C. Small EV-containing pellets were resuspended in 0.22 μm filtered HBS.

### Nanoparticle tracking analysis (NTA)

Concentration and size distribution of EVs were analyzed by NTA in a NanoSight NS500 unit (Malvern Instruments Ltd. Malvern, Worcestershire, United Kingdom), equipped with a 405 nm laser, a sCMOS camera and NTA 3.1 software. EV-enriched preparations were diluted for measurement at a concentration range around 10^9^ particles/ml (usually 1:1000), recommended for measurement by the instrument software. The settings used for data acquisition were as follows: camera level: 12, threshold: 10, capture: 60s, number of captures: 3, temperature: 25°C. These analyses were carried out in the laboratory of Dr. Hector Peinado, at the Spanish National Centre for Oncological Research (CNIO).

### Western blotting

Cells were lysed in lysis buffer (50 mM TRIS pH 7.5, 150 mM NaCl, 5 mM EDTA, 1% NP-40 (Millipore, Billerica, MA, United States) with protease inhibitors (leupeptin and pepstatin A at 1 µM). Lysates were incubated for 1 h on ice and centrifuged at 19,000 *x g* for 10 min at 4°C to discard nuclei. Protein content was determined by using Pierce Coomassie Plus (Bradford) assay kit (Thermo Scientific), following the manufacturer’s protocol, and the absorbance was measured at 595 nm using the Thermo Scientific™ Multiskan™ FC Filter-based Microplate Photometer (Thermo Scientific) combined with the SkanIt RE 4.1 software. 30–40 μg of protein were loaded on 10% SDS-PAGE gels (reducing conditions) and transferred to nitrocellulose membranes in a Trans-Blot Semi-Dry transfer cell with Trans-Blot Turbo Transfer Packs (Biorad). Membranes were blocked using 5% non-fat dry milk in PBS-containing 0.1% Tween 20 (PBS-T) for 1 h at RT. After washes with PBS-T, MICA and β-actin (housekeeping protein) were detected by incubating for 1 h at RT with the appropriate antibodies (listed in Supplementary Table S1). Washes were followed by incubation with either Alexa Fluor 790 streptavidin (0.4 μg/ml, Thermo Scientific) or streptavidin-horseradish peroxidase (HRP) (0.1 μg/ml, BioLegend) for 30 min at RT, or the secondary antibodies goat anti-mouse Ig (H + L) Alexa Fluor 700 (0.4 μg/ml, Thermo Scientific) or goat anti-mouse-HRP (0.8 μg/ml, Sigma), depending on the visualization method used. Proteins were visualized on the ChemiDoc MP Imaging Systems (Biorad) or on X-ray films, by using Amershman ECL Western Blotting Detection Reagent (GE Healthcare, Chicago, IL, United States), or the Odyssey Infrared Imaging System 9120 (LI-COR Biosciences, Lincoln, NE, United States), as indicated in the experiments.

For EV protein characterization, the same number of EVs (as determined by NTA) was used to compare treatments, although different cell lines produced different amounts of EVs. EV preparations were loaded in 10% SDS-PAGE gels under reducing conditions for MICA, Melan-A and β-actin detection, and in 12% SDS-PAGE gels under non-reducing conditions, for tetraspanin detection. Tetraspanins CD63, CD81 and CD9 were studied as EV markers. Goat anti-mouse Ig (H + L) Alexa Fluor 700 (CD63, CD81 and Melan-A detection) and Alexa Fluor 790 streptavidin (CD9 detection) were used as secondary antibodies. When possible, EVs secreted from CHO (Chinese-hamster ovary) cells transfected with MICA*008 [as described previously, (Ashiru et al., 2010)] were used as a positive control for MICA expression.

### EV characterization by ExoView^®^

EV characterization was performed by ExoView**®** R200 Platform (Unchained Labs, Pleasanton, CA, United States) using the human ExoflexTM kits (EV-TC-FLEX) in which chips were functionalised with anti-MICA antibody (MAB13002, R&D) and anti-ULBP2/5/6 antibody (AF1298, R&D) as capture antibodies following the protocol provided. The chips (*n* = 2) were pre-scanned using the provided protocol to identify any possible artefacts. For incubations, chips were carefully placed in 12-well plates, avoiding contact of the chip corners with the sides of the well. Ma-Mel-55 and H3122 EV-enriched preparations were diluted in the provided incubation solution buffer (final concentration indicated for each experiment in figure legends). 50 µL of the diluted sample were incubated on a functionalized chip without agitation overnight at RT. The following day, after several washes, 1 mg/ml of detection fluorescently labeled antibodies provided by the kit: anti-CD9 (CF^®^ 488A), anti-CD81 (CF5^®^555), and the anti-MICA antibody (MAB13002) labeled by Alexa Fluor^®^ 647 Conjugation Kit Fast (ab269823, Abcam, Cambridge, United Kingdom), were added to the chip and incubated for 1 h with gentle agitation. The chips were then washed and dried for analysis using ExoView^®^ platform and data later processed in ExoView Analyzer Software (Unchained Labs, Pleasanton, CA, United States). The fluorescence intensity threshold for each fluorescent antibody was stablished so that the percentage detected in the isotype spot was minimum.

### Statistical analysis

Analyses of significance were performed by using multiple t-tests in Graphpad Prism 8.0 software. *p*-values are shown only when the differences against the control DMSO are significant [*p* < 0.05 (*), *p* < 0.01 (**), *p* < 0.001 (***), *p* < 0.0001 (****)].

## Results

### Vemurafenib (BRAFi) and trametinib (MEKi), alone and in combination, modulate the surface expression of NK ligands on metastatic melanoma cells *in vitro*

**FIGURE 1:**
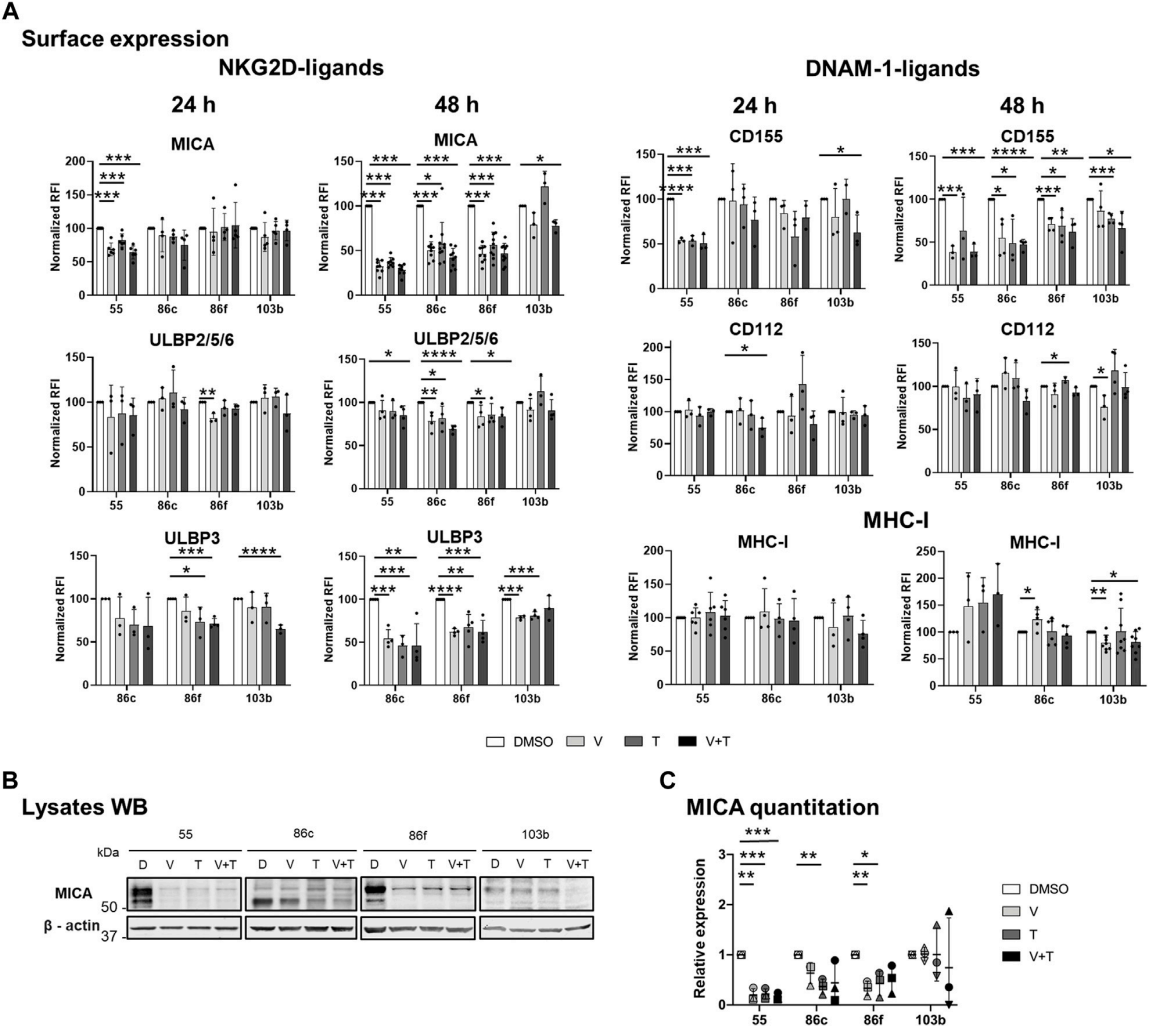
Effects of BRAFi, vemurafenib, and MEKi, trametinib, alone and in combination, on the expression of NK ligands in metastatic melanoma cells. The BRAF^V600E^-mutant (Ma-Mel-55 (55), Ma-Mel-86c (86c), Ma-Mel-86f (86f)) and BRAF-WT (Ma-Mel-103b (103b)) metastatic melanoma cells were treated *in vitro* with 1 μM vemurafenib (V), 50 nM trametinib (T), or both (V + T), for 24 h **(A)** or 48 h **(A– C)**. Control cells were treated with the carrier DMSO (D). **(A)**. Surface expression. Cells were analysed by flow cytometry. The Relative Fluorescence Intensity (RFI) was calculated as the Mean Fluorescence Intensity (MFI) for each antibody divided by the MFI of the isotype control (RFI = Antibody MFI/Isotype MFI). RFI values were then normalized to the expression of untreated cells (DMSO) [Normalized RFI = (Condition RFI/DMSO RFI)*100]. Each dot represents an independent experiment (n ≥ 3); bars show the mean ± SD. Ma-Mel-86f does not express surface MHC due to a β2m deletion. **(B)**. Western Blot (WB). 30–40 μg of protein were loaded on each lane of a 10% SDS–PAGE gels under reducing conditions. After transfer to nitrocellulose membranes, MICA was visualized by ECL, whereas ß-actin (used as a loading control) was detected using fluorescent Odyssey Infrared Imaging System 9120. The lower band corresponds with the glycosylphosphatidylinositol (GPI)-anchored MICA, which migrates faster in SDS-PAGE gels (Ashiru et al., 2013). A representative experiment is shown (*n* = 3). **(C)**. Quantitation of total MICA protein expression. The plot represents the MICA amount detected after different treatment conditions relative to the control (DMSO). WB band quantitation of three independent experiments was done using Image J. Horizontal bars indicate the mean and vertical bars the SD of the independent experiments, represented by different symbols (*n* = 3). Multiple t-tests were carried out in figures A, and C, and *p*-values are shown when the difference against the control DMSO is significant [*p* < 0.05 (*), *p* < 0.01 (**), *p* < 0.001 (***), *p* < 0.0001 (****)].

To evaluate the possibility of using immune ligands, such as NKG2D-L, to track immune competence during treatment with MAPKi, we studied in detail protein expression, both in cell surface and in their secreted material*,* after *in vitro* incubation with these drugs. First, the expression of several melanoma surface molecules, serving as ligands for NK cell activating receptors (NKG2D, DNAM-1), was analysed after 24 and 48 h of treatment with BRAFi, MEKi, or the combination thereof. The cell lines studied were Ma-Mel-55, Ma-Mel-86c, Ma-Mel-86f (all BRAF^V600E^ mutants) and Ma-Mel-103b (BRAF-WT). For each condition, cells were stained for several NKG2D-L (MICA, ULBP2/5/6 and ULBP3), DNAM-1 ligands (CD155 and CD112), and MHC class I, and analysed by flow cytometry. These cell lines do not express detectable levels of the NKG2D-L MICB, nor ULBP1, at the cell surface (López-Cobo et al., 2017), so these ligands were not studied here (Figure 1A).

Both inhibitors and their combination led to a decrease in surface MICA on BRAF^V600E^-mutant cell lines. A significant difference was observed for Ma-Mel-55, after 24 h, while for the rest of cell lines, significance was reached after 48 h of treatment, with around 50% reduction in the expression. ULBP2/5/6 expression also decreased in the three mutant cell lines, with a stronger effect at 48 h. BRAF-mutant lines with basal surface expression of ULBP3 (Ma-Mel-55 is negative for ULBP3) also showed a significant decrease when treated with single drugs or the combination for 48 h (Figure 1A).

DNAM-1 is another NK cell activating receptor that has a crucial role establishing the immune synapse. Thus, surface expression of its ligands, CD155 and CD112, was also analysed after MAPK inhibition. CD155 showed a significant decrease, in all the BRAF-mutant cell lines, after a 48-h culture with vemurafenib or with the combination treatment, while CD112 showed generally no significant decrease, even after 48 h (Figure 1A).

MHC class I, which can contribute to activation of CD8^+^ T cells but also to inhibition of NK cells, was slightly increased in MAPKi-treated cells, only significantly in the case of Ma-Mel-86c after 48 h of vemurafenib treatment. MEKi abrogated this effect, so downstream blockade of the route leads to different consequences (Figure 1A).

Total MICA protein after 48-h treatment was also analysed by Western Blot. In BRAF^V600E^-mutant cells, MICA intracellular protein correlated with surface expression after all the treatments tested (Figure 1A–C).

We compared data with the effect of the drugs in a BRAF-WT cell line. Interestingly, the BRAF-WT cell line Ma-Mel-103b, had different patterns to the mutant cells. In BRAF-WT cells, MEKi trametinib induced an increase in MICA, ULBP2/5/6, CD112 and MHC, although not statistically significant, and this effect was overruled when combined with BRAFi. In contrast, there was a significant decrease of surface ULBP3 and CD155 after incubation of BRAF-WT cells with MEKi, that was maintained in combination with BRAFi. Regarding MHC expression, while BRAF-mutant melanoma cell lines had an increase in surface MHC-I, it decreased in BRAF-WT cells after vemurafenib treatment [in line with (Frazao et al., 2017; López-Cobo et al., 2017)] and this decrease was maintained when both drugs were combined.

Altogether, in general in BRAF-mutant cells, MEKi and the combination of MEK and BRAF inhibition reproduced the trend previously described for the BRAFi vemurafenib (López-Cobo et al., 2017), decreasing NKG2D-L and DNAM-1-L at the cell surface. Our data suggest that downregulation of those molecules under MAPKi treatment might influence the drug resistance events observed in patients.

#### Release of soluble NKG2D-L by metastatic melanoma cells after MAPK inhibition *in vitro*

NKG2D-L can be released as soluble molecules after metalloprotease cleavage or in extracellular vesicles, which can modulate the immune response mediated by the NKG2D receptor. In fact, detection of these molecules in patient serum has been related with cancer progression (Wu et al., 2004; Paschen et al., 2009; Campos-Silva et al., 2022b). Therefore, it was important to test the effects of these drugs on extracellular secretion of each NKG2D-ligand. Theoretically, when analysing soluble NKG2D-L in biological samples, using immunoassays in which the capture and detection antibodies recognise the same protein, some signal could derive from EV-associated ligands. However, since NKG2D-L are very scarce proteins in EVs, this type of sandwich ELISA, capturing and detecting a single protein, usually does not detect much signal from EV-associated ligands (as observed in the next experiments). For simplicity, we will use the term *soluble proteins* when referring to the secreted proteins detected by a one-protein sandwich ELISA, as compared to *EV-released proteins* when capturing with an antibody directed to one protein and detecting with EV-associated markers.

**FIGURE 2:**
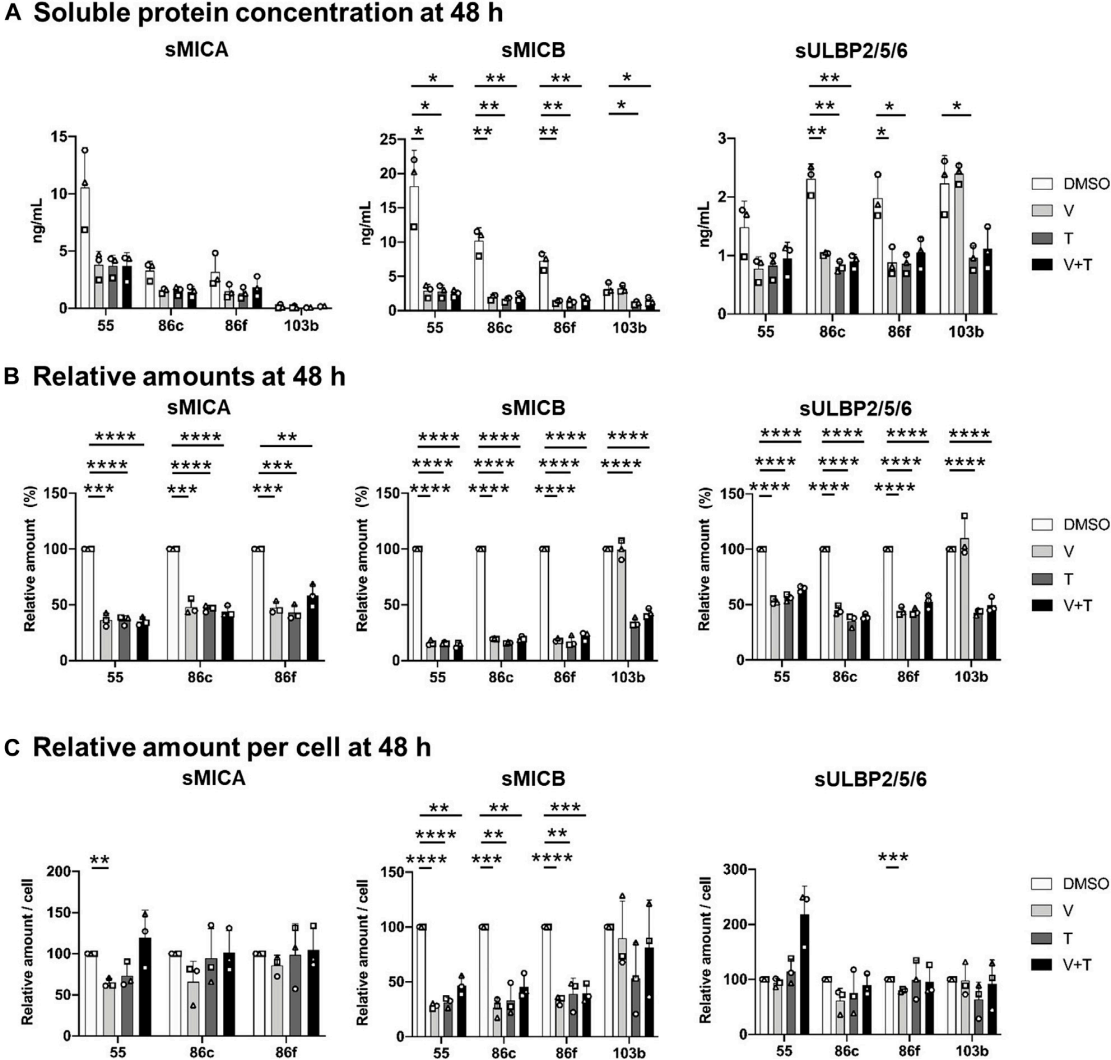
Effects of 48h-treatment with vemurafenib (BRAFi) and trametinib (MEKi), alone and in combination, on soluble NKG2D-L (sNKG2D-L). The BRAF^V600E^-mutant (Ma-Mel-55 (55), Ma-Mel-86c (86c), Ma-Mel-86f (86f)) and BRAF-WT (Ma-Mel-103b (103b)) metastatic melanoma cells were seeded in 12-well plates and treated with 1 μM of vemurafenib (V), 50 nM of trametinib (T), or both (V + T), for 48 h. Supernatants were collected and subjected to 200 *x g* centrifugation to discard floating cells. sNKG2D-L protein levels were measured by sandwich ELISA. The carrier DMSO was used as a control. **(A)**. Soluble protein concentration. Plots indicate the concentration (ng/ml) of sMICA, sMICB and sULBP2 secreted to the supernatant from each cell line at the different treatment conditions. **(B)**. Soluble protein in relative amounts. The amount of sMICA, sMICB and sULBP2 at the different treatment conditions relative to control cells (DMSO) is represented as percentage. The relative amounts of sMICA for Ma-Mel-103b cells are not represented in the plot due to low concentrations limiting the detection. **(C)**. Soluble protein relative amounts per cell. Plots represent the relative amount of sMICA, sMICB and sULBP2 at the different treatment conditions, as the ng/mL of soluble protein divided by the number of cells recovered in each condition, and then relativized to the control condition (DMSO). Data represent the mean ± SD in independent experiments (*n* = 3), depicted as different symbols. Multiple t-tests were performed to establish statistical significance (*p* < 0.05 (*), *p* < 0.01 (**), *p* < 0.001 (***), *p* < 0.0001 (****)).

Melanoma cells were treated with vemurafenib (BRAFi) and trametinib (MEKi), alone or in combination, for 24 and 48 h. At these time points, supernatants were collected, and soluble NKG2D-L (sNKG2D-L) were analysed by sandwich ELISA using antibodies for each particular protein (Figure 2; Supplementary Figure S1). Since treatments reduced cell proliferation differentially (except vemurafenib in the BRAF-WT cells, Ma-Mel-103b) (Supplementary Table S2), the concentration obtained directly in the supernatant was also analysed relative to untreated cells (Figure 2B), as well as to the cell number obtained at the end of the culture (Figure 2C).

24- and 48-h treatment with vemurafenib, trametinib, and the drug combination induced a significant decrease in the total amounts of soluble MICA, MICB and ULBP2/5/6 secreted from BRAF-mutant cells, Ma-Mel-55, -86c, and -86f, in comparison to the control condition, more significant after 48 h (Figure 2A, B; Supplementary Figure S1A, B). However, different results could be observed in BRAF-WT Ma-Mel-103b cells after treatment with different MAPK inhibitors. As expected, because vemurafenib does not target WT BRAF, this drug did not induce any effect on sNKG2D-L in BRAF-WT cells. Interestingly, the trend to increase surface MICA observed on these cells treated with MEKi, was not accompanied by the release of more sMICA, in fact, the concentration of sMICA was very low for this cell line, which would be beneficial for patients due to an increased recognition by NKG2D at the cell surface without receptor blockade. Moreover, trametinib, alone and in combination with vemurafenib, led to a significant decrease of sMICB and sULBP2/5/6 both after 24 and 48 h of treatment, also reflecting a loss of soluble protein despite the trend to increase or be maintained at cell surface.

Different concentrations could be observed for each soluble ligand (Figure 2A; Supplementary Figure S1A). Interestingly, the highest soluble amounts secreted to the media were noted for sMICB. This ligand is characterized for high levels of shedding, which together with the high internalization rate, could also explain its low cell surface expression. Soluble ULBP1 and ULBP3 were not detected in supernatants; ULBP1 is not expressed in these melanoma cells [(López-Cobo et al., 2017) and data not shown], while ULBP3 is mainly recruited to EVs, which makes detection usually more difficult by ELISA testing soluble protein.

NKG2D-L released per cell were, in general, not affected by MAPK inhibition. Only sMICB decreased per live cell after 24-h and 48-h of MAPKi treatment in BRAF-mutant cells (Figure2C; Supplementary Figure S1C). We can conclude that the decrease in total soluble MICA and ULBP2/5/6 after MAPKi treatment of BRAF-mutant and WT-cells is due to loss of cell proliferation and viability in drug-sensitive cells, rather than direct effects altering NKG2D-L secretion. However, the secretion of sMICB seems to be reduced by these treatments. All these observations would suggest a benefit for the patient, at the beginning of treatment, when mutant cells stop proliferation or die and immune blockade caused by sNKG2D-L would decrease. However, the decrease of activating ligands on the cell surface would favor immune escape of tumour cells.

### Differential recruitment of MICA to extracellular vesicles (EVs) after *in vitro* treatment of BRAF^V600E^-mutant metastatic melanoma cells with MAPK inhibitors

**FIGURE 3:**
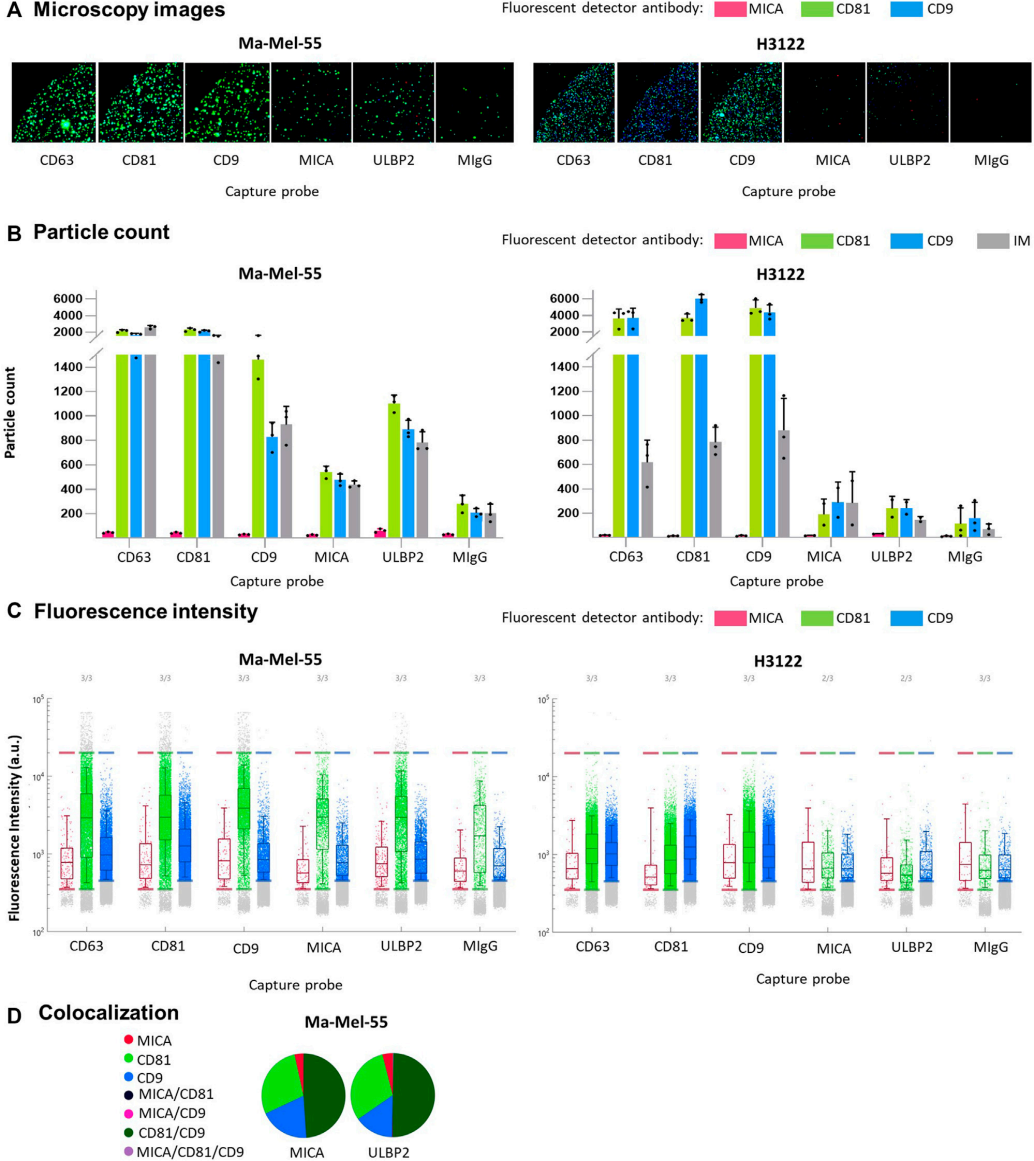
Single vesicle characterization of EV-enriched preparations. Ma-Mel-55 and H3122-derived EVs were diluted 1:2000 to a final concentration of 5∙10^5^ EV/μL (particle counts measured by NTA), incubated overnight in ExoFlex^®^ microchips functionalized with anti-MICA and anti-ULBP2 capture spots, together with anti-tetraspanin spots. Washed microchips were stained with a mix of fluorescent anti-MICA, anti-CD81 and anti-CD9 antibodies and analysed by ExoView^®^. **(A)**. Microscopy images: a representative fluorescence microscopy image from each capture probe is shown (*n* = 3). Green fluorescence corresponds to anti-CD81 detection antibody, blue fluorescence corresponds to anti-CD9 detection antibody and red fluorescence corresponds to anti-MICA detection antibody. **(B)**. Bar plots: particle counts for each fluorescent detection antibody and interferometry measurement (IM) on each capture probe. Each dot corresponds to a replicate; the mean and the SD are represented as bars. **(C)**. Fluorescence Intensity: dot plots represent each fluorescent dot detected for each capture probe/spot. The *Y* axes represent the fluorescence intensity for each detected fluorescent dot. The fluorescence intensity threshold for each fluorescent antibody was established so that the percentage detected in the isotype spot was minimum. **(D)**. Colocalization: pie charts represent the percentage of each single fluorescent antibody, as well as co-localized fluorescent antibodies detected for the capture probes anti-MICA and anti-ULBP2.

NKG2D-L released from cells in extracellular vesicles are generally more potent modulators of the NKG2D receptor on immune cells (Ashiru et al., 2010; Fernandez-Messina et al., 2010). We previously described the presence of MICA in EV-enriched fractions, however, no information on single vesicle was available yet. Thus, we used Exoview^®^, a technique that combines single particle interferometric reflectance imaging with antibody staining on microchips, for the characterization of protein content per vesicle. To demonstrate MICA expression at single vesicle level, Ma-Mel-55-derived EVs, known to contain MICA, were analysed, using as negative control EVs derived from the lung cancer cell line H3122. First, we characterized EV preparations from untreated cells by Nanoparticle Tracking Analysis (NTA) and WB (Supplementary Figure S2A, B). MICA was not detected in H3122-derived EVs by WB. Even though a minimal expression of MICA in these EVs cannot be ruled out, it would be lower than that of Ma-Mel-55-derived EVs in all cases. Exoview^®^ technology allowed to confirm the presence of the NKG2D-ligands MICA and ULBP2/5/6 in Ma-Mel-55-derived EVs at single vesicle resolution, and revealed the co-localization of both ligands in the same vesicle, as well as with CD81 and CD9 (Figure 3). Capture of MICA^+^ vesicles yielded lower signals than capture with tetraspanin antibodies, confirming the relative abundance of these proteins in EVs. In addition, MICA^+^ EVs from this melanoma cell line contained more CD81 than CD9 (Figure 3). EVs captured by anti-MICA antibody were successfully detected by fluorescent anti-tetraspanin antibodies, while fluorescent detection of MICA was rather low, highlighting that detection of EV-associated MICA is easier in combination with tetraspanins due to the relative abundance of these proteins.

**FIGURE 4:**
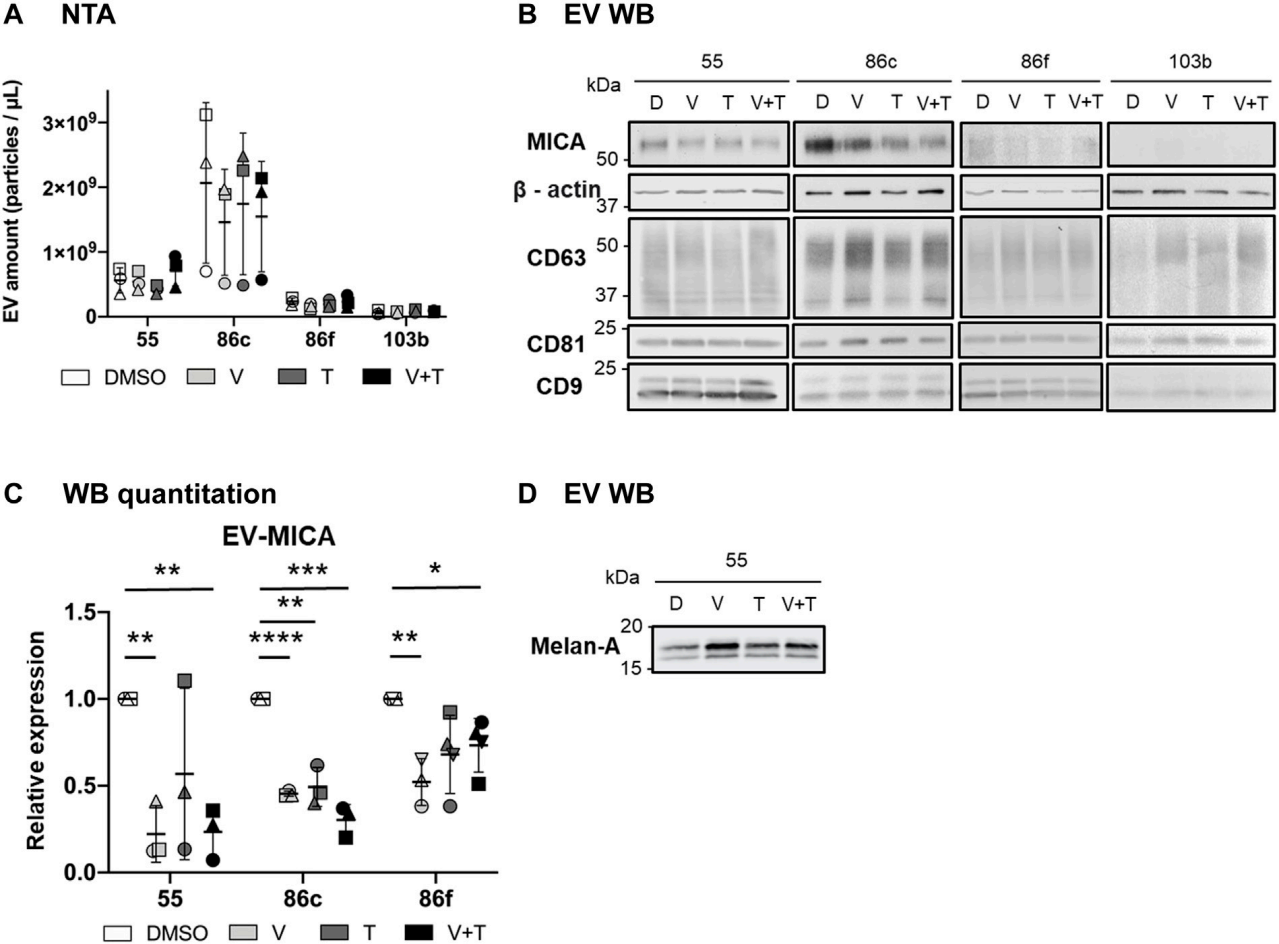
Effects of vemurafenib (BRAFi) and trametinib (MEKi), as *solo* or combined treatments, on EV-associated MICA. The BRAF^V600E^-mutant (Ma-Mel-55 (55), Ma-Mel-86c (86c), Ma-Mel-86f (86f)) and BRAF-WT (Ma-Mel-103b (103b)) metastatic melanoma cells were treated with either 1 μM of vemurafenib (V), 50 nM of trametinib (T), or both (V + T), for 48 h. As a control, cells were treated with the carrier DMSO (D). After treatment incubation, supernatants were collected and subjected to differential centrifugations for EV enrichment. **(A)**. EV analysis by Nanoparticle Tracking Analysis (NTA). The figure shows the concentration (particles/µl) of EV-enriched preparations at the different treatment conditions from each cell line. Horizontal bars indicate the mean and vertical bars the SD of the independent experiments, represented by different symbols (n = 3). **(B)**. Western Blot (WB). After EV enrichment, the same amount of EVs from each treatment condition was loaded on each lane of an SDS-PAGE (9.8 x 10^9^, 10 x 10^9^, 6.6 x 10^9^ and 1 x 10^9^ EVs from 55, 86c, 86f and 103b cells, respectively). After transfer to nitrocellulose membranes, MICA, and ß-actin (used as a loading control) were detected under reducing conditions, whereas non-reduced samples were used for CD63, CD81 and CD9 detection. ß-actin and tetraspanins were detected by using the Odyssey Infrared Imaging System 9120, and MICA was visualized by ECL. A representative experiment is shown (n = 3). Longer exposure times were needed for protein detection in Ma-Mel-86f and -103b samples. **(C)**. Relative EV-MICA protein expression. WB band quantitation was done with ImageJ software. The plot indicates the expression of MICA in EVs after the different treatment conditions relative to the control (DMSO). EV-MICA expression for Ma-Mel-103b cells is not represented in the figure due to low protein detection. Horizontal bars represent the mean and vertical bars the SD of the independent experiments, represented by different symbols (n = 3). Multiple t-tests were performed for the statistical analysis in figures A and C (*p* < 0.05 (*), *p* < 0.01 (**), *p* < 0.001 (***), *p* < 0.0001 (****)). **(D)**. Western Blot (WB). Equal amounts (measured by NTA) of Ma-Mel-55-derived EVs were loaded in 10% SDS-PAGE under reducing conditions. After transfer to nitrocellulose membranes, Melan-A was visualized using the Odyssey Infrared Imaging System 9120 (n = 1). This membrane was also used for detection of other markers in Figure 4 B (Ma-Mel-55-derived EVs).

Since quantification of EV-associated NKG2D-L is challenging to perform directly in supernatants, we initially characterized the effects of BRAFi and MEKi, alone and in combination, on EV-released MICA, separated from the soluble form by sequential centrifugations of melanoma cell conditioned media. To distinguish between soluble NKG2D-L proteins, that is, cleaved by metalloproteases, from EV-associated molecules, several precautions were taken. Although, in general, for EV analyses, we cannot rule out that protein aggregates could still be found in the 100 k pellet, in the case of MICA, soluble (metalloprotease cleaved) and EV-bound biochemical forms of the protein can be differentiated based on their size by WB, as we established in previous publications (Ashiru et al., 2010; Ashiru et al., 2013). Additionally, immunocapture experiments, involving different combinations of capture (MICA) and detection (tetraspanins) antibodies, were used to confirm MICA association to vesicles in the EV-enriched preparations obtained by ultracentrifugation, as previously shown. So, EV-enriched preparations obtained from melanoma cells treated during 48 h with MAPKi were characterized by different techniques as recommended by the ISEV. First, NTA was used to test size and EV concentration (Supplementary Figure S3A), revealing an average diameter of 150–200 nm, corresponding with small EVs. EV concentrations varied depending on the cell line, reproducing different growing rates and cell sizes. Despite this, the total EV concentration did not show statistically significant differences after the treatments in each cell line (Figure 4A). To characterize EV-associated MICA after treatment of metastatic melanoma cells with different MAPKi, the same number of EVs from each condition were analysed by WB (Figure 4B). Thus, these data show MICA per EV, rather than changes in total EV fractions. CD63, CD81 and CD9 were tested as EV markers correlating, in general, with ß-actin, indicating that MAPKi did not affect significantly tetraspanin expression. Treatment of Ma-Mel-55, -86c, -86f with vemurafenib, trametinib, and both therapies in combination, induced a decrease in EV-MICA protein in comparison to the control condition (Figure 4B, C). Ma-Mel-86f cells secreted very low amounts of EVs to the media (as measured by NTA; Figure 4A; Supplementary Figure S3A), however, MICA associated with EVs could be detected after long exposure times.

The BRAF-WT cell line, Ma-Mel-103b, also secreted very low amounts of EVs to the supernatant (Figure 4A, B), but in this case no signal could be detected for MICA. Thus, we can conclude that MICA secretion was low in BRAF-WT melanoma both in EVs, and as soluble protein (as shown in Figure 2). Increasing cell number or trying to increase EV recovery was not sufficient to detect a good signal.

All these results suggest that variations in NKG2D-L protein expression after MAPK inhibition could be reflected in the secretion to the extracellular milieu. However, it is important to remind that the heterogeneity observed in different cell lines could occur simultaneously in a patient with multiple melanoma lesions. For example, Ma-Mel-86c and -86f were derived from the same patient. Thus, testing NKG2D-L to follow immune evasion events would need to be analysed in a personalized manner. In addition, due to the complexity of the NKG2D system, the analysis of their different biochemical forms (surface, soluble proteins or EV-associated) would be needed for the complete understanding of the immune activation vs. evasion events in each patient.

**FIGURE 5:**
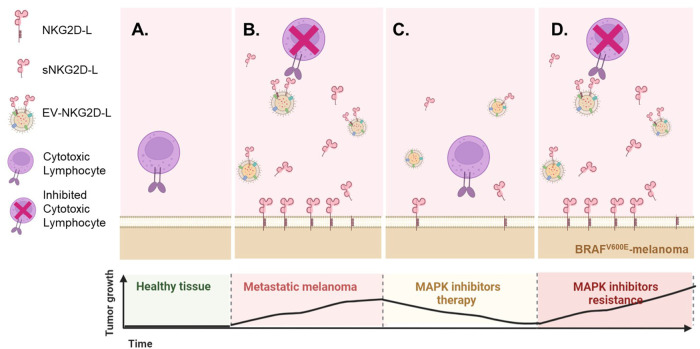
Possible scenarios of the NKG2D system as a consequence of MAPK inhibition. Based on the *in vitro* experiments shown above and in other published studies (Frazao et al., 2020), it is possible to model different situations in terms of NKG2D-mediated tumour recognition and evasion mechanisms due to soluble ligand blockade. However, little is known about the *in vivo* or *ex-vivo* circumstances. **(A).** Healthy melanocytes do not express NKG2D-L, while transformed and stressed cells usually present these activating immune ligands at the cell surface. **(B).** *In vitro*, metastatic melanoma cell lines express NKG2D-L both at the cell surface and in their soluble fraction, while MAPKi treatment reduce the levels of these ligands in both fractions **(C)**. A decrease in soluble NKG2D-L could be understood as a beneficial effect, since the cytotoxic lymphocytes will be available for attack mediated through the NKG2D receptor, but the decrease of the ligands at tumour cell surface will impede NKG2D-mediated recognition and allow their escape. **(D)**. Upon drug resistance, tumour proliferation is usually restored, which has been described to upregulate again the expression of NKG2D-L, most likely returning to the scenario previous to MAPKi treatment. However, the ideal scenario for a cancer patient would be a high expression of these ligands at the cell surface accompanied by low soluble levels. NKG2D-L may provide information about the immune status of the patient after MAPKi. Since, after MAPKi, changes in Melan-A (MART-1) were detected in EVs, combining information of EV-recruited NKG2D-L together with melanoma antigens could provide information on the possibility to activate several arms of the immune response. This figure was created using BioRender.

Cancer patients could present several scenarios regarding NKG2D biology. Based on the data obtained *in vitro*, we propose a model (Figure 5) considering surface and released NKG2D-L proteins, at different time points of melanoma treatment with MAPKi. In general, healthy melanocytes do not express NKG2D-L. However, when a tumour is proliferating, these ligands are expressed at the cell surface and are also released to the extracellular medium, which could represent an immune evasion mechanism. *In vitro*, MAPKi downmodulated NKG2D-L expression at the cell surface of BRAF^V600E^ tumour cells, and secreted ligands also decreased. In addition, previous reports show that drug resistance and tumour proliferation recover NKG2D-L expression, also increasing soluble ligands (Frazao et al., 2020). Therefore, soluble NKG2D-L could be analysed to investigate the capacity to eliminate NKG2D-L-expressing tumours by immune effector cells or as potential biomarkers of tumour relapse, especially during MAPKi therapy.

Previous reports show that, in several melanoma cells, expression of differentiation antigens (Tyrosinase, Melan-A or CSPG4) increased after short-term treatment with BRAFi followed by a decrease at long-term, affecting T cell recognition (Pieper et al., 2018; Harbers et al., 2021). Thus, we investigated whether the amount of tumour antigen Melan-A also changed in EVs from Ma-Mel-55 cells treated with BRAFi, MEKi, and the combination. Indeed, we could demonstrate for the first time the increase of Melan-A released in EVs after 48 h of treatment with MAPKi (Figure 4D), which could mirror the described tumour cell surface increased expression (Pieper et al., 2018). These data suggest that analyzing soluble and EV-associated NKG2D-L and tumour antigens can provide information on molecules responsible for the activation of several arms of the immune response. Because all these processes are dynamic, changing when cells acquire resistance, it will be important to understand what is the overall patient situation at every time point of treatment, so that the appropriate drug combination strategy is selected.

### Soluble and EV-associated NKG2D-L can be detected in metastatic melanoma patient sera and plasma during MAPKi treatment

**FIGURE 6:**
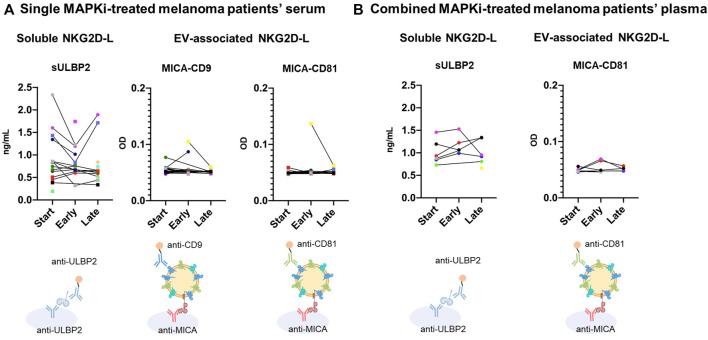
Soluble and EV-associated NKG2D-L in serum and plasma of metastatic melanoma patients at different time points of MAPKi treatment. Sera and plasma from metastatic melanoma patients treated with MAPKi were obtained at different time points of the treatment: start; early (1–2 months); late (3–20 months). Samples were diluted ½ in PBS-1% casein and tested by ELISA. For sULBP2 detection, a conventional sandwich ELISA using capture and detection antibodies against ULBP2 was used, as indicated. Concentration in ng/mL, calculated with standard curves of recombinant protein, is shown. No replicas of soluble protein experiments could be carried out due to sample volume limitations. For EV analysis, serum samples were incubated O/N at 4°C to avoid metalloprotease activity. Anti-MICA was used for capture and either biotin-conjugated anti-CD9 or anti-CD81 for detection, as indicated. Optical Density (OD) of the reaction product is shown in the *Y* axis. A representative experiment out of 2 is shown in figure A, although no more replicas could be carried out due to plasma volume limitations in figure **(B)**. Patient samples were obtained at Clínica Universidad de Navarra. Each patient is represented by different symbols and colors in all graphs. **(A)**. Single MAPKi-treated melanoma patients’ serum. Sera from a group of metastatic melanoma patients (Table 1) treated with single BRAFi/MEKi were analysed. **(B)**. Combined MAPKi-treated melanoma patients’ plasma. Plasma from a different group of metastatic melanoma patients (Table 2) treated with combined MAPKi were analysed.

As demonstrated by previous *in vitro* studies, the analysis of sNKG2D-L in biological samples from metastatic melanoma patients during MAPKi therapy could provide interesting information on the dynamic events leading to tumour immune evasion. Again here, we will use the term *soluble proteins* when detecting proteins using a one-protein sandwich ELISA, as compared to *EV-released proteins* when capturing with an antibody directed to one protein and detecting with EV-associated markers. As a first approach to study whether detection of released NKG2D-L, both soluble and EV-associated, could be evident in biological samples, a pilot study of sera from 22 metastatic melanoma patients and six non-cancer donors (demographic and clinical data are available in Table 1) was done. We first analysed samples obtained from patients at different time points during MAPKi treatment and tested soluble NKG2D-L by single protein sandwich ELISA. Most patient samples presented higher signals of sULBP2 than non-cancer samples (Supplementary Figure 4A). Within patients with highest sULBP2 concentration, a trend to decrease after 1–2 months of treatment (early time points) followed by an increase at later time points (3–4 months of treatment or later) was observed (Figure 6A). These data could reflect events of tumour recognition vs. tumour evasion fluctuating during treatment. No remarkable changes or trends could be observed for sMICA and sMICB levels (Supplementary Figure S4A). Next, we compared data of soluble NKG2D-ligands with EV-released. Importantly, in order to minimize EV subpopulation selection during sample processing for EV enrichment and to encourage easy translation in clinical settings in the future, direct detection by sandwich ELISA was performed (Campos-Silva et al., 2021; Campos-Silva et al., 2022a). To ensure the association of NKG2D-L to EVs, different combinations of capture and detection antibodies were used as depicted. Total EVs were tested as combinations of tetraspanins: CD63/CD9 and CD63/CD81 (Supplementary Figure S4B). As expected, levels of tetraspanins varied among different individuals, either patients with cancer or another condition. In any case, cancer patients presented, in general, higher CD63^+^-CD81^+^ signal than non-cancer donors, in line with previous observations in lung cancer patients compared with healthy donor plasma (Campos-Silva et al., 2022a). Then, we compared EV-released MICA (combining MICA capture with tetraspanin detection) with soluble released ligands. The detection of EV-associated MICA in patient sera was in general low, most likely due to the limits of detection in non-processed samples. Thus, only samples with high content of MICA released in EVs were detected. However, the signal was specific because samples with MICA^+^ EVs did not correspond with those with more CD63^+^-CD9^+^ or CD63^+^-CD81^+^. Moreover, signal was higher in these samples than in MICA^+^ EVs from Ma-Mel-86c (4 x 10^7^ EV/μL were loaded as a positive control compared to the negative control, 4 x 10^7^ EV/μL from H3122) (Supplementary Figure S4B). Only in three patient sera, EV-associated MICA was detected in the circulation, decreasing in two of them at later time points of MAPKi treatment, which could be reminiscent of the results obtained *in vitro* in drug-sensitive cells (Figure 6A (yellow and green dots) and Supplementary Figure S4B). sMICA samples did not coincide with MICA-EV positive, indicating that different biochemical forms could be detected preferentially in different samples and donors (Supplementary Figure S4B).

In summary, these results show that soluble and EV-associated NKG2D-L can be detected independently in patient sera, highlighting the need to understand the association of each different biochemical form with any clinical event. Moreover, changes in their levels could be observed during MAPKi treatment: at early-stages (1–2 months after treatment initiation), the levels of free and EV-bound protein decreased in certain patients; however, this effect was reverted in some cases for soluble ligands at later stages (after 3 months).

In an attempt to explore the effect of MAPKi combination in patients, serum and plasma samples from seven metastatic melanoma patients, obtained at different time points in a clinical trial, were analysed (demographic and clinical data are available in Table 2).

Plasma samples showed the same results as serum for soluble ligands (data not shown), while EV-associated MICA was only detected in plasma samples. In the case of soluble ligands, the concentrations for sMICA and sMICB were at the limit of detection (data not shown). Regarding sULBP2, patient 35 presented higher levels at early time points, that disappeared in the next time points [Figure 6B (purple dots) and Supplementary Figure S5A)]. However, patients 31 and 37 showed a considerable increase at later time points (9 and 7 months respectively) (Figure 6B (red and black dots) and Supplementary Figure S5A). These differences could be explained by the fact that P35 had a complete response and no signs of progression at later stages, while P31 and 37 presented partial responses and progression at the latest time points, when sULBP2 levels were increased. Specificity of MICA^+^ EVs signal was confirmed again comparing with CD63^+^-CD81^+^ EVs (Supplementary Figure S5B). Only patient 35 showed MICA^+^-CD81^+^ EVs at early time points (1 month after starting the treatment) (Figure 6B (purple dots) and Supplementary Figure S5B), that disappeared in the next time points (2, 5, 9 and 13 months after starting the treatment).

The results obtained in this pilot study provide evidence that these NKG2D-L can be detected in biological samples from melanoma patients, either as soluble and EV-associated, and that changes in concentration can occur at different time points during MAPKi treatment. This supports the importance of studying the whole fluctuations occurring in the NKG2D system before obtaining general conclusions; not all ligands behave similarly and soluble and EV-associated fractions can differ. These analyses could be of great relevance to inform about the immune competence of the patient. However, further analyses, studying large cohorts of patients should be performed to investigate the possible use of soluble or EV-associated NKG2D-L as biomarkers for MAPKi resistance events.

## Discussion

Targeted therapies and immunotherapies are used nowadays as first-line treatment for patients with advanced melanoma. Combined BRAF and MEK inhibition elicits a rapid response in 68% of patients with advanced melanoma harboring *BRAF* ^*V600E/K*^ mutation followed, however, by tumour relapse after a median of 11 months (Robert et al., 2019). Thus, although MAPKi has improved OS, it is important understanding drug resistance events and how targeted therapies can modulate the immune system [reviewed in (Kuske et al., 2018)]. Here we confirmed that the inhibition of MAPK leads to the modulation of several molecules that affect NK immune response and that BRAF-mutant melanoma cell lines, sensitive to vemurafenib, respond to the combination of BRAF and MEK inhibitors in a very similar manner to single inhibition. In particular, the activating immune ligands for NKG2D decreased clearly from cell surface after MAPK inhibition. We also provide the first single vesicle analysis of EV-associated MICA and further describe the effect of MAPK inhibition on soluble and EV-released NKG2D-L: in most cases, cell surface modulation was paralleled in total released NKG2D-L. Since NKG2D-L can be detected in patient sera, we propose that these molecules could be used to follow immune competence on melanoma patients. We present, to the best of our knowledge, the first pilot study testing soluble and EV-associated NKG2D-L in parallel that reveals changes on the levels of these molecules during patient treatment with MAPKi.

NKG2D-L are very well-known stress molecules that strongly activate NK cell cytotoxic activity against tumours when expressed at the cell surface. Because NKG2D is also present in all CD8^+^ T cells, NKG2D-L can also co-stimulate T cell response (Campos-Silva et al., 2022b). We previously reported that the BRAF^V600E^ inhibitor vemurafenib can affect surface expression of NKG2D-L on BRAF-mutant melanoma cells, leading to a decrease on NK recognition (López-Cobo et al., 2017). Here we show that MICA, ULBP2/5/6 and ULBP3 also decreased after treatment with the MEK inhibitor trametinib, and the combination of both drugs, during 48 h *in vitro*. DNAM-1 ligands, which are required for immune synapse formation, and MHC molecules can also be modulated by these drugs. However, all these effects show a degree of heterogeneity when comparing different BRAF-mutant cell lines, suggesting that different metastatic melanoma lesions may not trigger the same arms of the immune response. Because tumour heterogeneity can arise in different lesions within the same patient, it was important to study a number of cell lines. In our case, Ma-Mel-86c and -86f derived from the same patient but from different metastatic lesions were analysed, one of them with a defect on MHC-I expression due to a deletion on β2-microglobulin (β2m). Additionally, a non-BRAF-mutant melanoma cell line (Ma-Mel-103b) was also studied in parallel, to identify the possible effects of vemurafenib, alone or in combination, on cells lacking the targeted mutation. Strikingly, in some cases, BRAFi induced moderate expression changes on immune ligands in the BRAF-WT cell line, probably due to a paradox activation of the MAPK pathway by vemurafenib described in BRAF-WT cells (Holderfield et al., 2014). Other reports also show changes on cell surface NKG2D-L after melanoma treatment with MAPKi. In fact, when cells become resistant and recover proliferation, the expression of NKG2D-L is recovered (Frazao et al., 2020).

Besides the effect on patients, data presented here could help understanding NKG2D-L regulation. A highly complex regulation has been reported for the expression of NKG2D-L [reviewed in (Raulet et al., 2013)]. NKG2D-L expression has been related with proliferation (Molinero et al., 2003; Venkataraman et al., 2007) and with MAPK signalling involving the translation initiation factor eIF4e regulated by RAS and PI3K (Liu et al., 2012; Wu et al., 2012), which could explain the effect of ERK inhibition both in BRAF-mutant and non-mutant cells. In fact, a very important level of NKG2D-L regulation is the release of surface molecules either as cleaved soluble molecules or in EVs. This work also shows that BRAFi and MEKi, as *solo* or combined treatments *in vitro*, decreased soluble MICA, MICB and ULBP2 in supernatants from BRAF-mutant melanoma cells. However, this effect might also be largely due to the decrease in cell number, as cell proliferation decays. In addition, EV-associated MICA was also downregulated after the treatment with MAPKi in BRAF-mutant cells. This effect was not due to lower amounts of cells in treated cultures or lower amount of released EVs, but because of a reduced recruitment of MICA protein to each vesicle, correlating with the decrease observed at cell surface level. These data indicate that MAPKi-caused NKG2D-L decrease at the cell surface is not caused by an increased release to the supernatant. We have previously demonstrated *in vitro* that soluble and, in particular, EV-associated NKG2D-L impaired NK cell function (Ashiru et al., 2010; Fernandez-Messina et al., 2010). In addition, high amounts of soluble NKG2D-L in serum from cancer patients have also been associated with a downmodulation of the NKG2D receptor on NK immune effector cells, as well as NK function and this correlated with cancer progression [(Wu et al., 2000), for review, (Campos-Silva et al., 2022b)]. Thus, because soluble NKG2D-L are known to block NKG2D-mediated immune activation, data showing a decrease of secreted proteins suggest that MAPKi would not cause a general blockade of NK cells. If patients under these treatments have reduced circulation levels of NKG2D-L, this would imply a theoretical benefit. However, lower expression at the cell surface would help tumours to evade immune recognition, so an increase on surface activating ligands would be required, suggesting new pharmacological approaches, such as drugs increasing surface NKG2D-L, but blocking their release. As discussed in Figure 5, the ideal scenario for the patient would be a high NKG2D-L expression at the tumour cell surface, for immune recognition, accompanied by low amounts of released ligands.

In order to preliminarily explore any correlation between surface and released NKG2D-L for liquid biopsy, EV-MICA was analysed for the first time in parallel to soluble MICA, MICB and ULBP2 in sera from 29 melanoma patients, obtained at different time points after beginning the treatment with BRAFi, MEKi, combined targeted therapies or placebo in a clinical trial. Several patients showed a decreasing trend of sULBP2 levels at the beginning of the treatment, increasing at later time points. MICA-containing EVs were only detected in sera from five patients out of 29. In some cases, high soluble MICA was detected but not EV-associated MICA (P17) and *vice versa* (P13). This probably reflects that certain MICA alleles are preferentially recruited to EVs while others are not, which could depend on allelic differences or metabolic state of the tumour. In addition, detection of EV-MICA using anti-MICA antibody sandwich ELISA (designed for soluble protein detection) is usually low, as shown with Ma-Mel-86c-derived EV preparations, in which MICA could be observed in combination with CD9 or CD81, but was rather low in sMICA assays (Supplementary Figures S4, S5). This result evidences that most studies assaying soluble NKG2D-L most probably did not detect EV-associated NKG2D-L and thus, separate tests should be done for a complete data set. In fact, one patient presented a decrease in EV-MICA while soluble ligands remained constant (P13).

Although no conclusions on the suitability of MICA-EV and sNKG2D-L as biomarkers can be drawn from such a small number of melanoma patients, these preliminary results show that some patients had a decrease in serum sNKG2D-L at short-term after starting MAPKi treatment (patients 6, 8, 9, 10, 13, 16, 17 and 20), in line with the *in vitro* results, while the ligands increased, in certain patients at later time points (patients 6 and 16). In the group of melanoma patients treated with combined BRAF and MEK inhibitors, MICA^+^-EVs were detected only in one out of six patients and sULBP2 showed again a decrease after starting the treatment in one patient while an increase at later time points in other two patients, corresponding with time points when disease progression was detected in those two patients. It is tempting to speculate that the late-stage increase in NKG2D-L after long-term treatment could be associated to resistance development and tumour relapse. Indeed, a recent study reported an increase in cell surface and soluble NKG2D-L expression by melanoma cell lines after resistance development to MAPKi *in vitro,* together with increased susceptibility to NK killing (Frazao et al., 2020). In the same paper, no significant differences in sMICA were found in sera from 18 melanoma patients at the beginning of treatment compared to 6 months after starting targeted therapy. However, other sNKG2D-L or EV-associated molecules were not analysed. Recently, ERK5 activation has been described as another resistance mechanism to BRAF/MEK inhibitors used in clinics, suggesting alternative combinations (Tusa et al., 2018; Benito-Jardon et al., 2019; Cook and Lochhead, 2022). The effects of the activation or inhibition of this MAPK on NKG2D-L is another matter of interest for future studies.

The data presented here suggest that combination of MAPK inhibitors does not solve the immune evasion problems caused by single kinase inhibitors on NK biology, indicating that combination with other type of drugs might be more beneficial. We previously suggested that histone deacetylases (also used as single drug treatment in cancer and known to upregulate NKG2D-L) could represent an alternative for combination; sodium butyrate was able to restore NKG2D-L expression on melanoma cells when combined with vemurafenib *in vitro*, rescuing NK cell activation (López-Cobo et al., 2017). Interestingly, the order in which both drugs were administered was important in order to recover MICA surface expression. Several preclinical and clinical studies have shown that different epigenetic approaches, such as DNA methyltransferase inhibitors (decitabine) and HDACi (such as sodium butyrate) successfully complement BRAFi therapy (Lai et al., 2012; Zakharia et al., 2017; Gallagher et al., 2018). Currently, combination of targeted therapies with immunotherapies seems also a promising approach and different clinical trials are underway (Yu et al., 2019; Ziogas et al., 2021).

All these data together with reports showing that drug resistance could recover NK cell recognition (Frazao et al., 2020), contrast with the initial enhancement of the T cell response followed by T cell evasion at longer times (after 2–3 weeks of MAPKi treatment) (Pieper et al., 2018). Thus, early evasion of NK cell tumour recognition could constitute a failure in immune surveillance. This might facilitate changes in expression of other tumour antigens important for T cell recognition. Here we describe for the first time that the modulation of Melan-A (MART-1) observed at the cell surface of MAPKi-resistant cells can also be tracked in EVs. A complete characterization of the immunomodulatory effects of targeted therapies appears thus essential for the design of novel drug combination strategies.

In conclusion, even though combined BRAFi and MEKi targeted therapy leads to a delay in the appearance of drug resistance in melanoma patients, the *in vitro* study presented here shows that the combination therapy has similar consequences for NK cell recognition than single MAPK inhibition and does not avoid the emergence of immune evasion mechanisms related with NK immune activating ligands. The decrease on surface NKG2D-L, after BRAF-mutant melanoma cells *in vitro* treatment with vemurafenib and trametinib, was paralleled with a decrease in soluble and EV-associated proteins and suggested the evaluation of these biochemical forms in patient sera to follow up the evolution of immune modulating ligands and the reversal described by others at drug resistance. However, the potential role for NKG2D-L as biomarkers of the immunomodulatory effects of MAPKi targeted therapies, will need to be studied in a personalised manner, analysing all ligands, which was highlighted by the heterogeneity observed in patient samples during MAPKi treatment. However, large cohorts of patients should be analysed to investigate the potential of NKG2D-L as biomarkers for the clinics of MAPKi-treated metastatic melanoma patients. This study further provides the basis to support the combination with other therapeutic agents that could increase surface NKG2D-L and block their release to the supernatant.

## Data Availability

The original contributions presented in the study are included in the article/Supplementary Material, further inquiries can be directed to the corresponding author.
