# Supplementary material for: MAPK inhibitors dynamically affect melanoma release of immune NKG2D-ligands, as soluble protein and extracellular vesicle-associated
Source: Front Cell Dev Biol. 2023 Jan 16;10:1055288. doi: 10.3389/fcell.2022.1055288 (PMC9884675; doi:10.3389/fcell.2022.1055288)
Supplement: Supplementary file 1 [file Table1.pdf]

Supplementary table 1. Antibodies used.

Antibody	Identifier	Concentration	Source
**Flow cytometry**			
**Mouse monoclonal anti-MICA**	MAB13002, isotype IgG1	0.02 µg/µl	R&D systems, Minneapolis, MN, USA
**Mouse monoclonal anti-ULBP2/5/6**	MAB1298, isotype IgG1	0.02 µg/µl	R&D systems, Minneapolis, MN, USA
**Mouse monoclonal anti-ULBP3**	MAB1517, isotype IgG1	0.02 µg/µl	R&D systems, Minneapolis, MN, USA
**Mouse monoclonal anti-CD115**	D171, MA5-13493, isotype IgG1	0.02 µg/µl	Thermo Scientific, Waltham, MA, USA
**Mouse monoclonal anti-CD112**	B-C12, isotype IgG2b	0.02 µg/µl	Santa Cruz Biotechnology, Dallas, TX, USA
**Mouse anti-MHC**	HP1F7, isotype IgG1	0.02 µg/µl	Described in Pérez-Villar et al., 1997
**Mouse monoclonal IgG1 isotype control**	MOPC21	0.02 µg/µl	Sigma-Aldrich, St. Louis, MO, USA
**Mouse monoclonal IgG2b isotype control**	MPC11	0.02 µg/µl	Sigma-Aldrich, St. Louis, MO, USA
**Western Blot**			
**Mouse monoclonal anti β-actin**	AC-15	0.13 µg/ml	Sigma-Aldrich, St. Louis, MO, USA

**Mouse monoclonal anti-CD81**	M-38	1 µg/ml	Kind gift from Vaclav Horejsi
**Biotinylated goat monoclonal anti-CD9**	VJ1/20	1 µg/ml	Immunostep S.L, Salamanca, Spain
**Mouse monoclonal anti-CD63**	Tea3/18	1 µg/ml	Immunostep S.L, Salamanca, Spain
**Biotinylated goat polyclonal anti-MICA**	BAF1300	0.4 µg/ml	R&D systems, Minneapolis, MN, USA
**Rabbit polyclonal anti-calreticulin**	NB600	4 µg/ml	Novus Biologicals, Englewood, CO, USA
**Mouse anti-Melan-A**	MAB8008	0.5 µg/ml	R&D systems, Minneapolis, MN, USA
**ELISA (soluble protein)**			
**Capture**			
**Mouse monoclonal anti-MICA**	MAB13002	5 µg/ml	R&D systems, Minneapolis, MN, USA
**Goat polyclonal anti-MICB**	AF1599	5 µg/ml	R&D systems, Minneapolis, MN, USA
**Goat polyclonal anti-ULBP1**	AF1380	5 µg/ml	R&D systems, Minneapolis, MN, USA
**Goat polyclonal anti-ULBP2/5/6**	AF1298	5 µg/ml	R&D systems, Minneapolis, MN, USA
**Goat polyclonal anti-ULBP3**	AF1517	5 µg/ml	R&D systems, Minneapolis, MN, USA
**Detection**			
**Biotinylated goat polyclonal anti-MICA**	BAF1300	0.4 µg/ml	R&D systems, Minneapolis, MN, USA

**Biotinylated goat polyclonal anti-MICB**	BAF1599	0.4 µg/ml	R&D systems, Minneapolis, MN, USA
**Biotinylated goat polyclonal anti-ULBP1**	BAF1380	0.4 µg/ml	R&D systems, Minneapolis, MN, USA
**Biotinylated goat polyclonal anti-ULBP2/5/6**	BAF1298	0.4 µg/ml	R&D systems, Minneapolis, MN, USA
**Biotinylated goat polyclonal anti-ULBP3**	BAF1517	0.4 µg/ml	R&D systems, Minneapolis, MN, USA
**ELISA (EV protein)**			
**Capture**			
**Mouse monoclonal anti-MICA**	MAB13002	6 µg/mL	R&D systems, Minneapolis, MN, USA
**Mouse monoclonal anti-CD63**	Tea3/18	6 µg/mL	Immunostep S.L, Salamanca, Spain
**Mouse monoclonal IgG1 isotype control**	MOPC21	6 µg/mL	Sigma-Aldrich, St. Louis, MO, USA
**Detection**			
**Mouse monoclonal anti-CD81**	M-38	0.4 µg/mL	Immunostep S.L, Salamanca, Spain
**Mouse monoclonal anti-CD9**	VJ1/20	0.2 µg/mL	Immunostep S.L, Salamanca, Spain

Supplementary table 2. Mean of the total amount of melanoma cells recovered after 48 h of incubation with DMSO, vemurafenib, trametinib or the combination (V+T).

		DMSO	Vemurafenib	Trametinib	V+T
**$BRAF^{V600E}$**	Ma-Mel-55	$21.5 \cdot 10^6$	$18.6 \cdot 10^6$	$19.7 \cdot 10^6$	$16.1 \cdot 10^6$
	Ma-Mel-86c	$34.8 \cdot 10^6$	$19.1 \cdot 10^6$	$16.5 \cdot 10^6$	$17.9 \cdot 10^6$
	Ma-Mel-86f	$9.1 \cdot 10^6$	$6.2 \cdot 10^6$	$7.1 \cdot 10^6$	$7.7 \cdot 10^6$
**BRAF-WT**	Ma-Mel-103b	$6.6 \cdot 10^6$	$6.4 \cdot 10^6$	$5.6 \cdot 10^6$	$5.6 \cdot 10^6$

Equal amounts of cells were seeded for each treatment condition (different numbers among cell lines depending on cell shape and size). Average of three experimental repetitions in 15 cm cell culture dishes is shown.

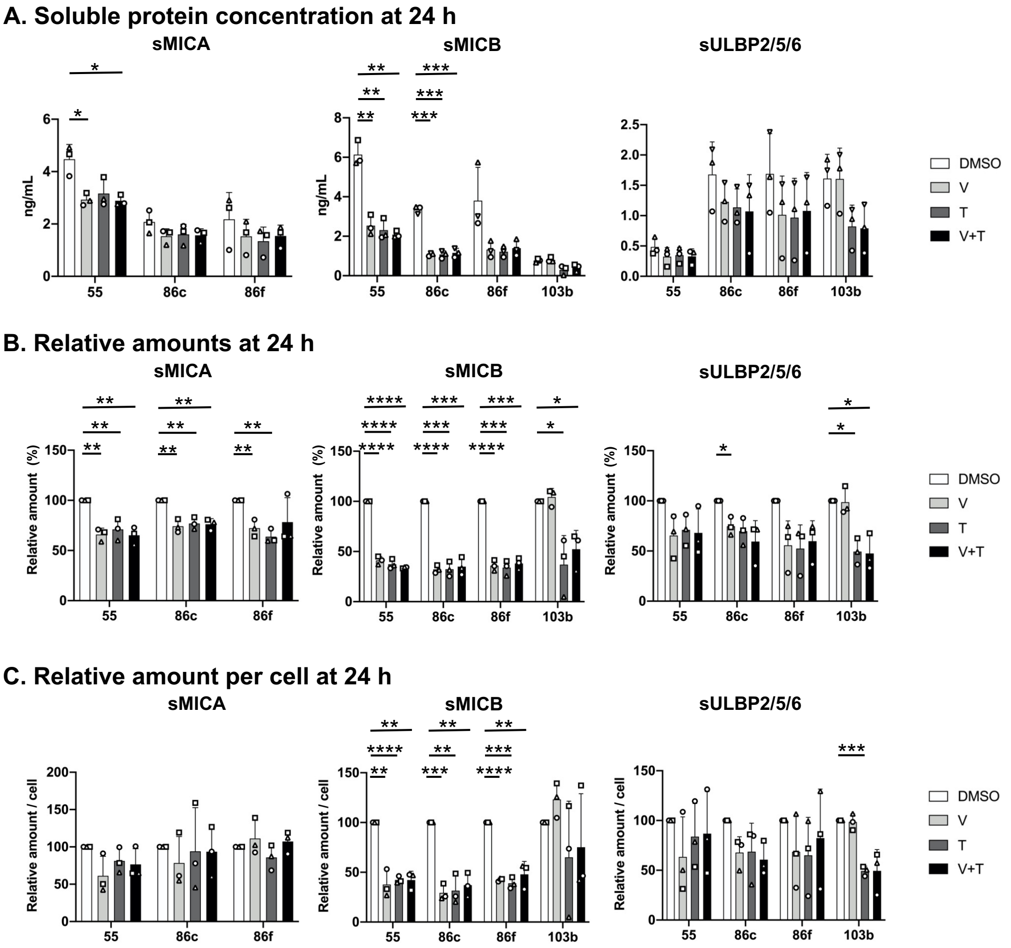

Supplementary figure 1. Effects of 24-h treatment with vemurafenib and trametinib, alone and in combination, on soluble NKG2D-L (sNKG2D-L). The $BRAF^{V600E}$-mutant (Ma-Mel-55 (55), Ma-Mel-86c (86c), Ma-Mel-86f (86f)) and BRAF-WT (Ma-Mel-103b (103b)) metastatic melanoma cells were treated with either 1 µM of vemurafenib, 50 nM of trametinib, or both (V+T), for 24 hours, in 12-well plates. DMSO was used as a vehicle control. After the treatment, supernatants were collected and subjected to 200 *x g* centrifugation to discard floating cells. Supernatants were then used for the study of soluble NKG2D-L protein levels by sandwich ELISA. **A. Soluble protein concentration.** Plots indicate the concentration (ng/mL) of sMICA, sMICB and sULBP2 secreted to the supernatant. No sMICA secretion was detected from Ma-Mel-103b cells, so this cell line is not included in the graph. **B. Soluble protein relative to untreated control.** Figures show the amount of sMICA, sMICB and sULBP2 in treatments relative to DMSO control and expressed as percentage (DMSO). **C. Soluble protein relative amounts per cell.** Plots represent the relative amount of sMICA, sMICB and sULBP2 at the different treatment conditions, as the ng/mL of soluble protein divided by the number of cells recovered in each condition, taking the control condition (DMSO) as 100%. Data show the mean ± SD in independent experiments (n=3), each one represented by a different symbol ($p < 0.05$ (*), $p < 0.01$ (**), $p < 0.001$ (***), $p < 0.0001$ (****)).

A. NTA

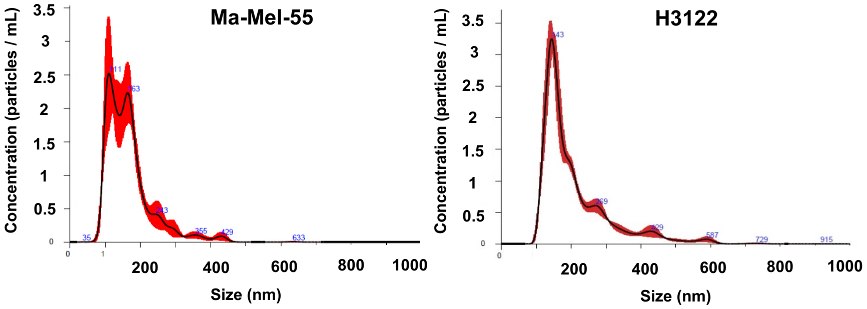

Average ⌀	165.8 +/- 4.2 nm
Mode ⌀	117.5 +/- 9.1 nm
Particles/µL	1.34×10^9

Average ⌀	208.2 +/- 2.6 nm
Mode ⌀	146.5 +/- 4.6 nm
Particles/µL	1.55×10^9

B. WB

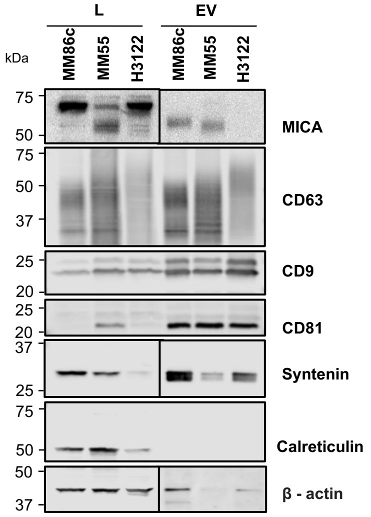

Supplementary figure 2. Characterization of EV-enriched preparations prior to single vesicle analysis. EV preparations derived from Ma-Mel-55 and H3122 cell lines were used for characterization. **A. Nanoparticle tracking analysis (NTA).** Average size and concentration of EVs were obtained in a NanoSight equipment capturing three videos of 60 s per measurement. ⌀: diameter. **B. EV characterization by Western Blot (WB).** 10^{10} EVs derived from Ma-Mel-86c, Ma-Mel-55 and H3122 cell lines were characterized by Western Blot. MICA, syntenin, calreticulin and ß-actin proteins were detected in reducing conditions; CD63, CD81 and CD9 in non-reducing conditions. SA or GAM secondary antibodies were visualized using Odyssey Infrared Imaging System 9120 or X-ray films (MICA). Calreticulin was detected as an endoplasmic reticulum resident protein not present in the EV fraction.

A. NTA

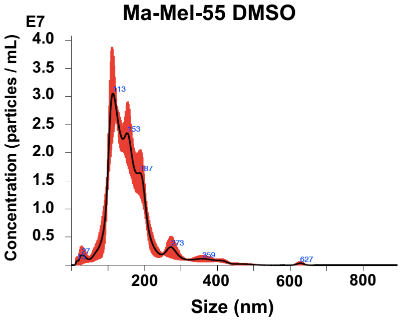

Average ⌀	159.3 +/- 5.4 nm
Mode ⌀	120.3 +/- 5.2 nm
Particles/μL	7.4 x 10^8

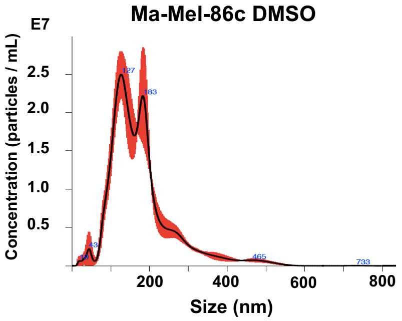

Average ⌀	177.0 +/- 3.2 nm
Mode ⌀	156.1 +/- 14.3 nm
Particles/μL	3.1 x 10^9

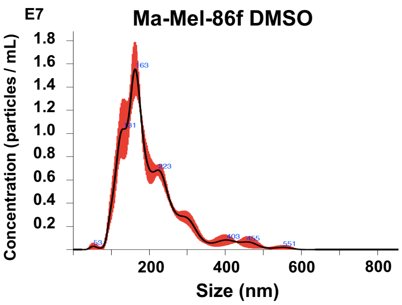

Average ⌀	200.5 +/- 4.7 nm
Mode ⌀	147.7 +/- 11.3 nm
Particles/μL	2.9 x 10^8

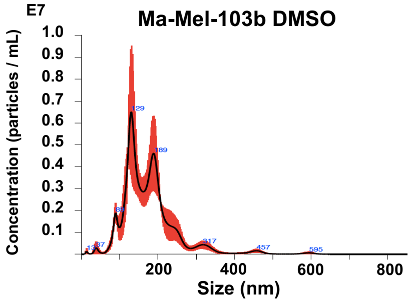

Average ⌀	174.1 +/- 6.7 nm
Mode ⌀	165.4 +/- 17.6 nm
Particles/μL	6.8 x 10^7

Supplementary figure 3. Characterization of EV preparations from MAPKi-treated cells. The $BRAF^{V600E}$-mutant (Ma-Mel-55, Ma-Mel-86c, Ma-Mel-86f) and BRAF-WT (Ma-Mel-103b) metastatic melanoma cells were treated with either 1 µM of vemurafenib, 50 nM of trametinib, or both, for 48 hours. The carrier DMSO was used as untreated control. After treatment incubation, supernatants were collected and subjected to differential centrifugations for EV enrichment. **A. Size and concentration analysis by Nanoparticle Tracking Analysis (NTA).** EV preparations from each condition were analyzed by NanoSight. A representative image for each cell line (Ma-Mel-55, -86c, -86f, and -103b) is shown. Mean and mode size, and average concentration (particles/µl) are listed in the table. ⌀: diameter.

Single MAPKi-treated melanoma patients' serum

A. Soluble NKG2D-L

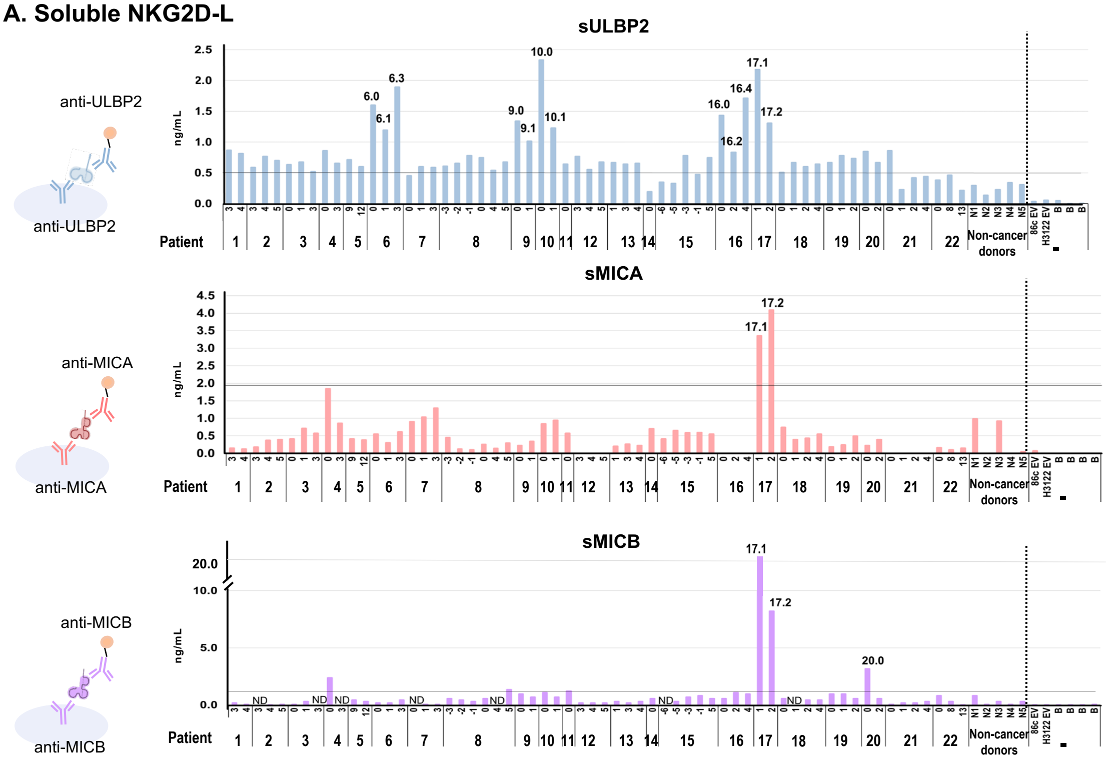

B. EV-associated NKG2D-L

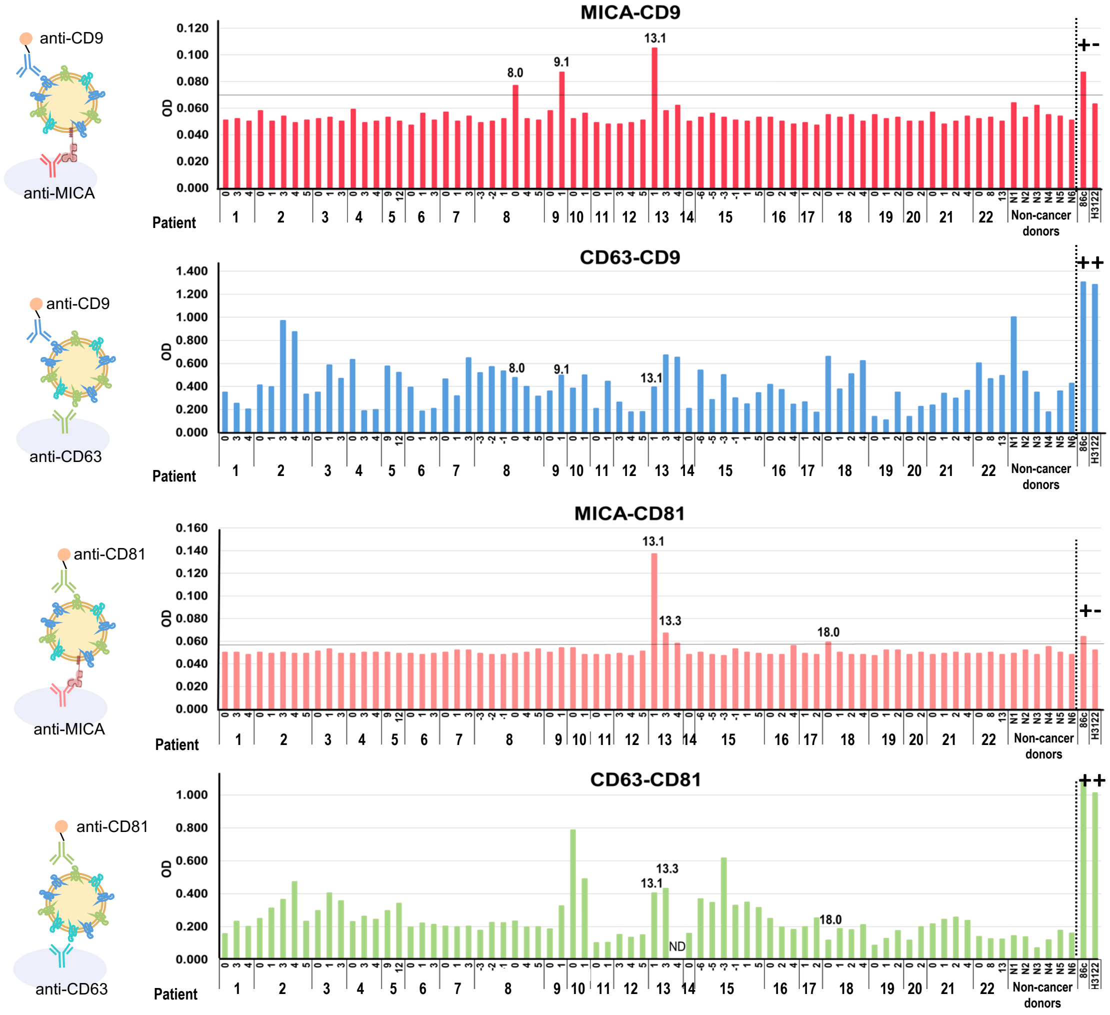

Supplementary figure 4. Soluble and EV-associated NKG2D-L in metastatic melanoma patients' sera treated with MAPKi. Metastatic melanoma patients' sera obtained at different time points during MAPKi treatment were diluted ½ in PBS containing 1% casein. Soluble NKG2D-L (**A**) and EVs (**B**) were tested by ELISA (for EV analysis, sample incubation was performed at 4°C to avoid metalloprotease activity). **A.** For sMICA, sMICB and sULBP2 detection, a conventional sandwich ELISA using capture and detection antibodies against the same protein were used, as indicated. Concentration in ng/mL, calculated with standard curves of recombinant protein, is shown for soluble proteins. No replica of soluble proteins experiments could be carried out due to sample volume limitations. **B.** For EV analysis, anti-MICA or anti-CD63 were used for capture and biotin-conjugated anti-CD9 or anti-CD81 for detection, as indicated. Optical Density (OD) of the reaction product is shown for EV-associated proteins. A representative experiment out of 2 is shown. The second repetition showed the same pattern with lower signals in general, most likely due to freeze-thaw cycles. No more replicas of this experiment could be carried out due to serum volume limitations. The threshold line for each NKG2D-L protein detection was calculated as the mean of the non-cancer donor (N1-6) signals plus 3 times the Standard Deviation (N6 was not assayed in soluble protein analyses due to volume limitations). X axis: patient number and months after starting MAPKi treatment (negative numbers indicate months before starting the treatment). Patient samples were obtained at Clínica Universidad de Navarra. ND: Non-Determined (due to sample volume limitations). B = blanks. Ma-Mel-86c- (86c) and H3122-derived-EVs (4 x 10^7 EVs/µL) were used as positive or negative controls, as indicated in the figure.

Combined MAPKi-treated melanoma patients' plasma

A. Soluble NKG2D-L

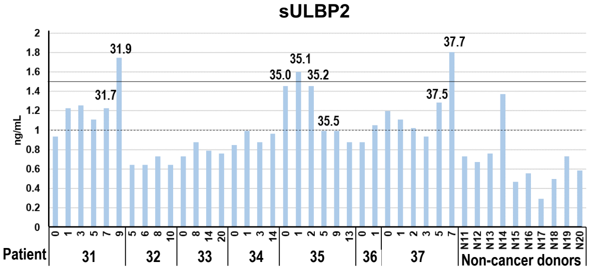

B. EV-associated NKG2D-L

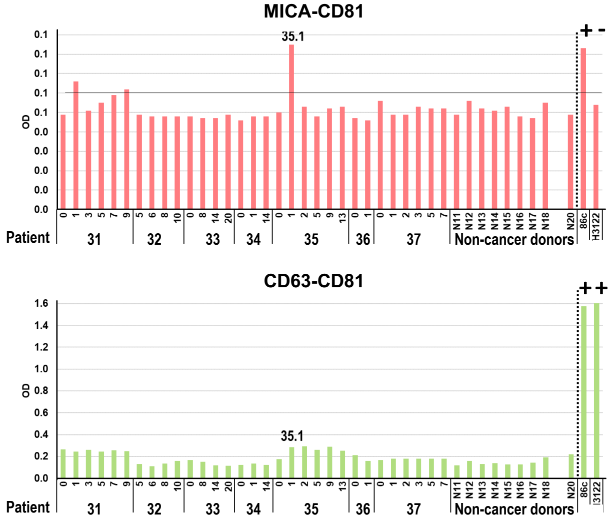

Supplementary figure 5. Soluble and EV-associated NKG2D-L in metastatic melanoma patients' plasma during treatment with MAPKi combination. Metastatic melanoma patients' plasma at different time points during MAPKi treatment were diluted ½ in PBS-1% casein and tested by ELISA. **A.** For sULBP2 detection, a conventional sandwich ELISA using capture and detection antibodies against ULBP2 were used. Concentration in ng/mL, calculated with standard curves of recombinant protein, is shown for sULBP2. **B.** For EV analysis, anti-MICA or anti-CD63 were used for capture and, and biotin-conjugated anti-CD9 or anti-CD81 for detection, as indicated. Optical Density (OD) of the reaction product is shown for EV-associated proteins. The threshold line for each NKG2D-L protein detection was calculated as the mean of the non-cancer donor (N11-20) signals plus 3 times the Standard Deviation. A second threshold was calculated for sULBP2 because donor N14, had a high concentration. Since non-cancer donors were individuals that attended the hospital for a blood test, a higher NKG2D-L expression in one of them could be due to another pathology. MICA-CD9, sMICA and sMICB were also analysed but showed negative signals (at the limit of detection) for all patient samples. N9 plasma rendered abnormally high non-specific signals when detected with anti-CD81 or anti-CD9, so it was eliminated from EV analyses. X axis: patient number and months after starting MAPKi treatment. No more replicas of this experiment could be carried out due to plasma volume limitations. Patient samples were obtained at Clínica Universidad de Navarra. ND: Non-Determined (due to sample volume limitations). B = blanks. Ma-Mel-86c- (86c) and H3122-derived-EVs were used as positive or negative controls, as indicated in the figure.
